# Raspberry-like Nanoheterostructures Comprising Glutathione-Capped Gold Nanoclusters Grown on the Lanthanide Nanoparticle Surface

**DOI:** 10.1021/acs.chemmater.3c03333

**Published:** 2024-03-05

**Authors:** Irene Pérez-Herráez, Juan Ferrera-González, Elena Zaballos-García, María González-Béjar, Julia Pérez-Prieto

**Affiliations:** † Instituto de Ciencia Molecular (ICMol), Departamento de Química Orgánica, Universitat de València, C/Catedrático José Beltrán, 2, Paterna, Valencia 46980, Spain; ‡ Department of Organic Chemistry, Universitat de València, Av. Vicent Andrés Estellés s/n, 46100 Burjassot, Valencia ,Spain

## Abstract

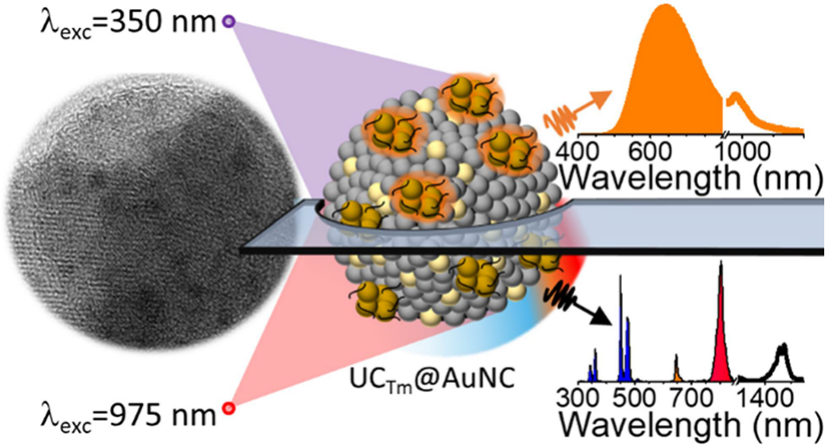

Bare lanthanide-doped nanoparticles (LnNPs), in particular, NaYF_4_:Yb^3+^,Tm^3+^ NPs (UC_Tm_), have been seeded in situ with gold cations to be used in the subsequent growth of gold nanoclusters (AuNCs) in the presence of glutathione (GSH) to obtain a novel UC_Tm_@AuNC nanoheterostructure (NHS) with a raspberry-like morphology. UC_Tm_@AuNC displays unique optical properties (multiple absorption and emission wavelengths). Specifically, upon 350 nm excitation, it exhibits AuNC photoluminescence (PL) (500–1200 nm, λ_max_ 650 nm) and Yb emission (λ_max_ 980 nm); this is the first example of Yb sensitization in a UC_Tm_@AuNC NHS. Moreover, under 980 nm excitation, it displays (i) upconverting PL of the UC_Tm_ (at the blue, red and NIR-I, ca. 800 nm, regions); (ii) two-photon PL of AuNC; and (iii) down-shifting PL of thulium (around 1470 nm). The occurrence of energy transfer from UC_Tm_ to AuNCs in the UC_Tm_@AuNC NHS was evidenced by the drastic lengthening of the AuNC PL lifetime (τ_PL_) (from few hundred nanoseconds to more than one hundred microseconds). Initial biological assessment of UC_Tm_@AuNC NHSs in vitro revealed high biocompatibility and bioimaging capabilities upon near-infrared excitation.

## Introduction

Inorganic nanoheterostructures (NHSs) combine different nanodomains, thereby leading to nanosystems with different functional properties, and consequently, they can perform different functions at the same time. There are several examples of functional inorganic NHSs.^1−3^ However, although lanthanide nanoparticles (LnNPs) and gold nanoclusters (AuNCs) have shown low toxicity, biocompatibility, and chemical and photochemical stability, the synthesis of functional NHSs combining both nanomaterials remains unexplored.

Undoubtedly, LnNPs are encouraging nanomaterials to develop functional photoactive NHSs due to their unique optical features.^4,5^ LnNPs can be excited with NIR light and display tunable multiple narrow upconversion and/or downshifting emissions and exhibit high thermal stability; moreover, they neither undergo photoblinking nor photobleaching.^6−9^ Ideally, their crystal lattice could be modified to favor the growth of AuNCs.

AuNCs are small nanoparticles (less than 3 nm in diameter) that have proved promising capabilities for applications in catalysis, sensing, or bioimaging.^10−12^ AuNCs exhibit remarkable properties, such as size-dependent photoluminescence (PL) with tunable emission colors, PL lifetimes in the order of ns, and large Stokes shift. These ultrasmall nanoparticles are nonplasmonic, in contrast to gold nanoparticles (AuNPs), and present molecule-like physicochemical properties, such as HOMO–LUMO transitions. AuNCs have been combined with other materials (e.g., carbon dots,^13^ MnO_2_,^14^ MoS_2_ nanosheets^15^ or metal–organic frameworks (MOFs),^16^ among others) to benefit the capabilities of both materials or their synergy.

The unique features of both materials (LnNPs and AuNCs) together with the photophysical crosstalk between both counterparts in the corresponding NHS, via energy transfer (ET) processes, may rise synergistic effects, but the exact mechanism(s) of how these energy transfer processes occur is still under debate.^17^

The combination of both materials has so far been limited to the synthesis of nanohybrids by using multistep synthetic protocols, for example, the conjugation of captopril-capped AuNCs (Au_25_(capt)_18_^–^) to a LnNP with a multiple core–shell heterostructure, namely, NaGdF_4_:0.3%Nd@NaGdF_4_@NaGdF_4_:10%Yb,1%Er@NaGdF_4_:10%Yb@NaNdF_4_:10%Yb.^18^ This nanohybrid emitted dual-mode luminescence (upconversion/downshifting) under 808 nm light excitation and, simultaneously, a thermal effect was observed. LnNPs acted as energy donors by means of the NIR-to-Vis upconversion process, while the conjugated AuNCs acted as acceptors in a resonant energy transfer (RET) process to produce singlet oxygen (^1^O_2_) upon 808 nm excitation. Meanwhile, the NIR-to-NIR downshifting emission was almost unaffected by the conjugated AuNCs.^18^ Two more examples of nanohybrids comprising downshifting core–shell LnNPs and AuNCs have been reported.^18−20^ Once again, the synthetic protocol required a three-step process, since both nanoparticles had to be previously synthesized before their combination through covalent bonding^18,20^ or electrostatic interaction.^19^

In this context, LnNPs can serve as an ideal platform to build a multifunctional NHS by following a cation exchange strategy^5,21,22^ to display unique photophysical features of interest for other technological applications, such as multicolor imaging upon different excitation wavelengths to differentiate probes that display different emissive colors and time-resolved photoluminescence imaging to distinguish between probes based on their different decay rates. In fact, due to the current great interest in long-lived emissive probes for photoluminescence imaging and sensing, new NIR-responsive upconversion nanohybrids have been designed to achieve lifetime lengthening of common emissive probes (e.g., fluorescein) from nanoseconds to microseconds.^23^ However, although the synthetic protocol can be simple, organic dyes are not as robust as AuNCs and consequently, a multifunctional NHS could fit better for this purpose.

In this respect, preparation of heterostructures has been reported, such as that in which perovskite quantum dots (PQDs) nanocrystals are embedded in a single LnNP nanocrystal^24^ The structural stability of the perovskite increased significantly, especially against polar solvents, and the system exhibited a dual mode luminescent behavior (exciting the perovskite or the LnNP) as well as resonant energy transfer from the UV upconversion emission to the perovskite.^24^ Other than that, heterostructured nanofibers composed of TiO_2_, LnNPs, and CdS QDs have also been reported. These 1D materials presented a wide spectral absorption that participated in resonance energy transfer processes (from the upconversion emission to the other counterparts) and exhibited enhanced photocatalytic properties under IR or solar irradiation.^4^

However, there are no NHS combining LnNP and AuNCs by means of a cation exchange strategy. Herein, we describe a strategy to synthesize a LnNP@AuNC NHS with a raspberry-like morphology, by growing glutathione-capped AuNCs via a preliminary cation exchange of β-NaYF_4_:Yb^3+^(24.9%) and Tm^3+^(0.3%) LnNP (UC_Tm_) surface cations with Au^3+^ ions, followed by a mild reduction in the presence of glutathione (GSH). This strategy simplified the synthesis of the UC_Tm_@AuNC NHS by avoiding the use of additional chemicals and complicated synthetic and purification steps. The NHS photophysical properties were recorded at different excitation wavelengths (350 and 980 nm) to explore the capabilities of each counterpart to act as an energy donor or acceptor. A preliminary cytotoxicity study of the UC_Tm_@AuNC NHS was also performed.

## Experimental Section

### Materials

The chemicals used for the synthesis of the UC_Tm_@AuNC nanoheterostructure were YCl_3_·6H_2_O, YbCl_3_·6H_2_O, and TmCl_3_·6H_2_O (>99.9% all of them) and 1-octadecene (95%), oleic acid (70%), and NaOH and NH_4_F (99.99%). Nitrosonium tetrafluoroborate (NOBF_4_), gold(III) chloride trihydrate (HAuCl_4_·3H_2_O), *L*-GSH, methanol, ethanol, chloroform, cyclohexane, acetone, *N,N*-dimethylformamide (DMF), phosphate buffer saline (PBS), 3-(4,5-dimethylthiazol-2-yl)-2,5-diphenyltetrazolium bromide (MTT), deuterated water, and acetonitrile were also used; all these chemicals were purchased from Sigma-Aldrich and used as received without previous purification. In all cases, the glassware used in the following procedures was cleaned in a bath of freshly prepared solution of HNO_3_–HCl (1:3, v/v) and rinsed thoroughly with water prior to use. For all aqueous solutions, high purity deionized water from the Millipore system was used.

### Synthesis

#### UC_Tm_@OA

The oleate-capped β-NaYF_4_:Yb^3+^(24.9%) and Tm^3+^(0.3%) LnNPs were synthesized by thermal decomposition with oleic acid and octadecene at high temperature following a well-known protocol.^25−31^ See further details in the Supporting Information.

#### UC_Tm_@BF_4_

A ligand-exchange strategy with NOBF_4_ was carried out to replace the original oleate ligands attached to the LnNPs as previously reported.^32,33^ See further details in the Supporting Information.

#### UC_Tm_

The functionalization with GSH was performed by stirring for 24 h an aqueous solution containing the peptide in excess and an aliquot of the UC_Tm_@BF_4_ dispersion (82 mg·mL^–1^ in DMF). The mixture was centrifuged (12,000 *g* for 15 min), and the precipitate was redispersed in water. Five centrifugation-redispersion cycles were performed. Finally, the UC_Tm_ was redispersed in deuterated water.

#### UC_Tm_@AuNC Nanoheterostructure

Briefly, to a colloidal dispersion of UC_Tm_@BF_4_ in DMF (100 μL, 15 mg·mL^–1^) a freshly prepared aqueous solution of HAuCl_4_ (500 μL, 50 mM) was added and the mixture was stirred at room temperature for 1 h in the dark. Then, an aqueous solution of GSH (500 μL, 100 mM) was added and stirred during another hour. After that, the resulting mixture was set (with no stirring or shaking) in the dark for a week, thereby obtaining a luminescent colloid under UV light. Then, the formed NHS was precipitated by the addition of acetonitrile in excess and isolated by centrifugation at 18,000 *g* for 15 min, according to a previously reported protocol for AuNCs.^34^ The pellet was purified three times by dispersing the UC_Tm_@AuNC in water (10 mL) and precipitating the NHS in acetonitrile (30 mL), followed by centrifugation (18,000 *g* for 15 min). Finally, purified UC_Tm_@AuNC was dispersed in deuterated water (pH 4.8).

#### Gold Nanoclusters

A freshly prepared aqueous solution of HAuCl_4_ (500 μL, 50 mM) was added to 100 μL of DMF and stirred at room temperature for 1 h. Then, an aqueous solution of GSH (500 μL, 100 mM) was added and stirred for another hour and then set in the dark for a week, thus obtaining a luminescent colloid (under UV light). The nanocluster was precipitated by addition of acetonitrile and isolated by centrifugation at 18,000 *g* for 15 min.^34^ The pellet was purified three times by dispersing the AuNC in water (10 mL) and then precipitating the AuNC in acetonitrile (30 mL) and centrifuging (18,000 *g* for 15 min). Finally, the purified AuNC was dispersed in deuterated water. The final concentration of AuNC dispersion was calculated by weight difference, by drying an aliquot of the previous sample.

#### Characterization

Centrifugation of the samples was carried out in a Thermo-Scientific Legend XIR. The supernatant was collected with care to avoid disturbing the precipitate. Transmission electron microscopy (TEM) images were acquired using a Jeol 1010 microscope operating at 100 kV equipped with a digital camera (AMT RX80; 8 megapixels) and a HITACHI HT7800 microscope with a filament of LaB_6_ operated at 100 kV. The samples were deposited on a Formvar/carbon film supported on a 300-mesh copper grid from dispersions in DMF and dried under vacuum at room temperature. High-resolution transmission electron microscopy (HRTEM) images were recorded using a TECNAI G2 F20 microscope operating at 200 kV (point resolution of 0.24 nm) and equipped with a CCD GATAN camera. Energy-dispersive X-ray analysis (EDAX) was performed in the TECNAI G2 F20 microscope by using a Si(Li) detector (active area: 30 mm^2^, resolution: <142 eV) and the Genesis software. X-ray diffraction (XRD) analyses were performed on a Bruker D8 Advance A25 diffractometer using Cu K_α_ (λ 1.54060 Å) radiation at a voltage of 40 kV and 30 mA and a LynxEye detector. The powder diffraction pattern was scanned over the angular range of 2–80° (2θ) with a step size of 0.020° at room temperature.

All Fourier transform infrared (FTIR) spectra were obtained using an FTIR Thermo Nicolet iS10 spectrophotometer at room temperature with 256 scans and a resolution of 4 cm^–1^ between 400 and 4000 cm^–1^. Thermal gravimetric analyses (TGA) were acquired with a TGA 550 from TA Instruments with an operative temperature range of 25–950 °C (rate of 10 °C·min^–1^) and under nitrogen flux. X-ray photoelectron spectroscopy (XPS) spectra were acquired with a VG-Microtech Multilab 3000 equipment, which has a semispherical electron analyzer with 9 channels, pass energy of 2200 eV, and an X-ray radiation source with Mg and Al anodes. Inductively coupled plasma-mass spectrometry (ICP-MS) analyses were carried out in triplicate using an ICP-MS Agilent 7900.

#### Photophysical Characterization

All measurements were carried out in deuterated water under air, unless otherwise indicated, for 0.5 mg/mL dispersions of UC_Tm_, AuNCs, and UC_Tm_@AuNCs. UV/vis/NIR absorption spectra were recorded in a PerkinElmer 1050+ UV/vis/NIR spectrophotometer. All data were acquired using 1 cm × 1 cm path length quartz cuvettes. Steady-state emission spectra and time-resolved kinetics were recorded in a FLS1000 photoluminescence spectrometer (Edinburgh Instruments) equipped with a 450 W ozone-free xenon arc lamp and coupled to a 2 W CW 980 nm laser diode (PSU-III-LED, CNI Optoelectronics Technology Co. Ltd.). The Fluoracle software was used to register the data and fit the kinetic traces. The detection correction was applied to all the spectral data. Laser power density (PD) reported for emission spectra was obtained by measuring laser power in the FLS1000 sample chamber with a thermal sensor (S470C, Thorlabs) coupled to a PM100D console (Thorlabs), and the rectangular laser spot profile was measured with a LT665 (Ophir) silicon CCD camera (D4σX and D4σY definitions which afforded an approximately 0.19 cm^2^ laser spot area).

The emission deactivation kinetics at 600 nm of the AuNC and UC_Tm_@AuNC NHS were obtained through laser-induced emission mode with the photomultiplier detector of a laser flash photolysis spectrometer (LP980-KS, Edinburgh Instruments) equipped with a Nd:YAG INDI Quanta-Ray laser (Spectra Physics). The excitation wavelength was at 355 nm with a pulse energy of 10 mJ. Additionally, a 395 nm-long pass filter was used before the detection monochromator. The kinetics were fitted with the L900 software (Edinburgh Instruments).

Absolute photoluminescence quantum yield (Φ_PL_) and upconversion quantum yield (UCQY) measurements were performed in sealed-tube quartz cuvettes (1 cm optical path length) in a Quantaurus QY Plus (C13534–11, Hamamatsu Photonics K.K.) coupled to a NIR photoluminescence measurement unit (C13684-01, Hamamatsu Photonics K.K.). The measurements were carried out under air atmosphere; the visible and NIR spectral range (400–1300 nm) was registered for both excitation wavelengths (355 and 980 nm). Two excitation sources were used: the built-in Xe lamp and a 2.5 W 980 nm continuous wave laser (MDL-III-980, CNI Optoelectronics Technology Co. Ltd.). The laser power was varied to characterize the UCQY using a variable neutral density disk. The reported excitation irradiance was obtained from the laser spot size provided by the manufacturer, and the laser power was measured with a power meter (PD300-3W, Ophir Optronics Solutions Ltd.) within the integration sphere.

Near infrared laser scanning microscopy (NIR-LSM) technique was performed using an Olympus FV1000MPE laser scanning confocal coupled to an Olympus BX61WI upright microscope equipped with a 25× water immersion objective (1.05 NA). This microscope was provided with a Mai Tai HP DeepSee femtosecond laser (pulse width 100–200 fs; repetition rate 80 MHz) as the excitation source. Images were acquired by means of appropriated emission filters, the indicated dwell time, and a resolution of 1024 × 1024 pixels. Emission is detected simultaneously in two detection channels (channel 1, C1:420–500; channel 2, C2:515–580 nm). Samples were prepared by drop-casting onto a 25 × 75 mm microscope glass slide, then covered with a 22 × 22 mm glass slide, and sealed when the solvent was evaporated. The excitation energy is expressed as the total energy density (fluence, *F*) delivered during the dwell time. It depends on the laser average output power (measured by the system), the excitation wavelength, the laser transmissivity of the acousto-optic modulator, the objective transmission, the objective numerical aperture, and the dwell time, according to an already reported calculation.^35^

#### In Vitro Experiments

Cervical cancer HeLa cells (ATCC, USA) from Central Service for Experimental Research (SCSIE) at the University of Valencia were grown in high glucose Dulbecco’s modified Eagle medium (DMEM, Gibco) containing 10% fetal bovine serum (FBS) and 1% antibiotics (100 U·mL^–1^ penicillin and 100 μg·mL^–1^ streptomycin) and 250 μg·mL^–1^ fungizone at 37 °C with 5% CO_2_ in a humified incubator.

Biocompatibility was evaluated at a low passage number (between 5 and 8) by using MTT colorimetric viability assay (Invitrogen) as the protocol. The possibility of contamination was excluded by regularly performing mycoplasma tests. Aqueous MTT was employed as a measure of cell survival percent after 24 h of incubation. For the experiments, 100 mL of suspended HeLa cells (1 × 10^5^ cell·mL^–1^; cell counting was carried out in an automated cell counter Cell Countess II from Invitrogen) was seeded in 96-well plates 24 h prior to the addition of UC_Tm_@AuNC or controls (AuNC or UC_Tm_ to create subconfluent cultures (80% confluency) as recommended for viability testing adapting the ISO 10993-5 guideline.

The culture medium was removed, and cells were treated with the UC_Tm_@AuNC NHS or controls (AuNC or UC_Tm_) at different dilution factors at serial concentrations of 50, 100, 150, 200, 250, 300, 500, and 800 μg·mL^–1^ for 24 h. Then, the medium was removed, and the cells were washed with PBS. Finally, the content of each well was replaced by 50 μL of MTT solution (5 mg·mL^–1^ in PBS) and incubated at 37 °C and 5.0% CO_2_ for 2 h. After this time, the absorbance was recorded at 570 and 650 nm (as a reference) in a well plate reader (Synergy H1, BioTekl) at 20 °C. All MTT assays were performed three times in triplicate. A negative control was also performed by exposing cells to the vehicle (culture medium with water at the same percentage of the samples). Untreated cells were exposed only to the culture medium, and positive controls were conducted by using H_2_O_2_ and tertbutyl hydroperoxide.

To examine the cellular uptake, cervical cancer HeLa cells were seeded in completed phenol-free culture media on culture dishes of 60 mm (ca. 2.5 × 10^4^ cell·mL^–1^) and incubated at 37 °C with 5% CO_2_ in a humified incubator for 24 h for cell adherence. Then, the media was replaced with fresh media (3 mL) containing UC_Tm_@AuNC NHS or controls (AuNC or UC_Tm_) at concentrations of 200 μg·mL^–1^ for 24 h. Prior imaging, the cells were washed with PBS and coincubated with 3 mL of fresh phenol red-free growth media containing Hoechst 33342 (Invitrogen) for 15 min. For fixation of the cells, upon nuclear staining with Hoechst, cells were incubated with 4% paraformaldehyde in PBS for 30 min at 37 °C and washed with PBS; fresh PBS was placed before imaging. The samples were visualized using the NIR-LSM technique^35^ with an Olympus FV100MPE coupled to a Mai-Tai HP Deep See (Spectra Physics) as the excitation source. Thus, LnNPs were excited at 975 nm and their emission was collected in C1 (420–500 nm) at 100 μs·pixel^–1^ dwell time (F 0.9 kJ·cm^–2^), while Hoechst 33342 was excited by two-photon excitation at 750 nm and its fluorescence was collected again in C1 (420–500 nm) at 4 μs·pixel^–1^ dwell time (F 37 J·cm^–2^).

## Results and Discussion

### Synthesis of the UC_Tm_@AuNC NHS

First, the synthesis of LnNPs, which served as platforms to grow AuNCs, was performed. Briefly, oleate-capped NaYF_4_:Yb^3+^,Tm^3+^ (UC_Tm_@OA) was synthesized by a thermal decomposition method using oleate and 1-octadecene (see Experimental Section).^25−31^Figure S1 shows the XRD pattern of crystalline uniform hexagonal prisms of β-UC_Tm_@OA and the TEM image with an average size of (28.4 ± 1.3) × (22.7 ± 1.2) nm. The atomic ratio of lanthanides in the nanoparticles was obtained by ICP-MS and was Y^3+^(74.8%):Yb^3+^(24.9%):Tm^3+^(0.3%). Then, a ligand exchange with NOBF_4_ was carried out to replace the original OA ligands attached to the LnNP surface^32,33^ by the labile BF_4_^–^ anions, thus leading to water-dispersible (hydrophilic) bare UC_Tm_@BF_4_. FTIR measurements revealed the disappearance of the C–H stretching signals of the −CH_2_– and −CH– groups of the oleate alkyl chain (2923 and 2851 cm^–1^) together with the vibrations of the bidentate coordination of the carboxylate group (1560 and 1457 cm^–1^, respectively)^36^ after treatment with NOBF_4_ (Figure S2).

**Figure 1:**
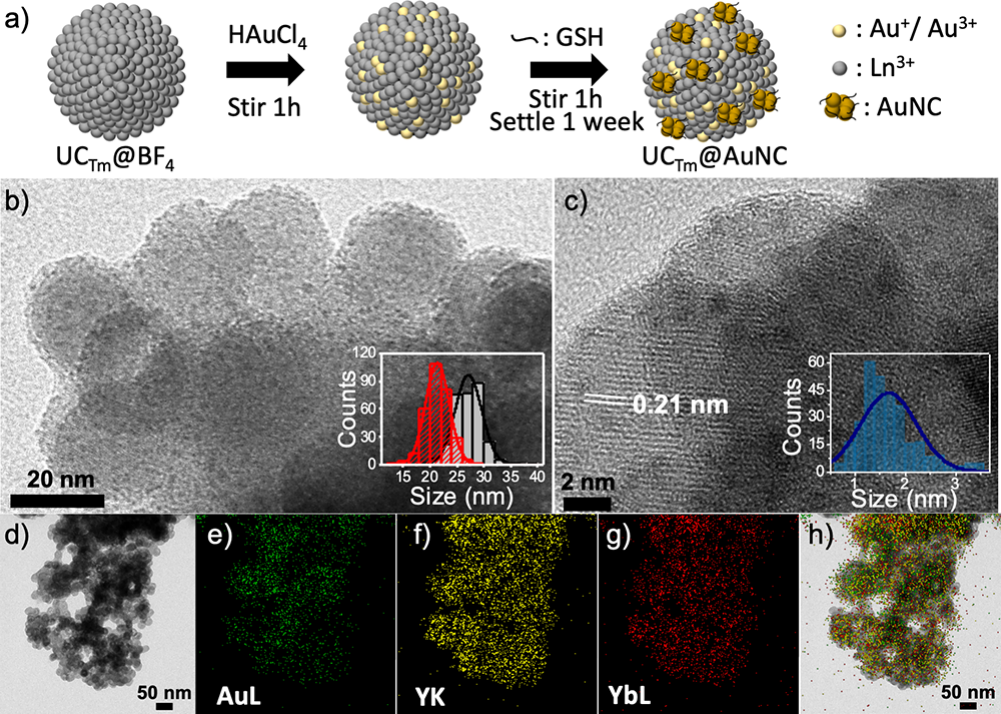
Structural characterization of UC_Tm_@AuNC NHS. (a) Schematic illustration of the synthesis of UC_Tm_@AuNC NHS. (b,c) HRTEM images of UC_Tm_@AuNC NHS (Insets: (b) length (black) and width (red) of UC_Tm_@OA and (c) diameter of AuNC). (d) HRTEM image of UC_Tm_@AuNC and the mapping signals of the same region for the (e) AuL, (f) YK, and (g) YbL. (h) Overlapped images 1d–1g.

The preparation of the UC_Tm_@AuNC NHS was accomplished by a very simple in situ AuNC synthesis protocol divided in three steps (Figure 1a): (i) cation exchange followed by (ii) a mild Au^3+^ reduction with an excess of GSH and (iii) size-focused growth of thermodynamically stable small AuNCs.

Cation exchange is a technique of note commonly used to synthesize multicomponent NHS.^37^ Their preparation is based on nanoscale cation exchange combined with postsynthetic heteroepitaxial seeded growth.^38^ Additionally, cation-induced aggregation of oligomeric Au(I)–thiolate complexes has been previously reported to occur when using certain multivalent cations, including some lanthanides (e.g., Sm^3+^, Y^3+^, Yb^3+^, and Ce^3+^) due to coordination between the cations and the carboxylate groups of GSH in the complexes to form inter- and/or intracomplex cross-links.^39,40^ As a result, the negative charge on the complexes is neutralized, and aurophilic bonds and dense aggregates are formed, eventually leading to AuNCs with aggregation-induced emission.^40^

All this considered, the cation exchange process consisted of simply mixing a concentrated aqueous solution of Au^3+^ (a freshly prepared aqueous solution of HAuCl_4_) with an aliquot of UC_Tm_@BF_4_ dispersion in DMF followed by stirring in the dark for 1 h. Despite the simplicity of the Au^3+^ pretreatment of UC_Tm_, this seems to be of paramount importance to grow AuNCs on the UC surface. After pretreatment with Au^3+^ for 1 h and centrifugation (20,000 *g* for 20 min), the solid was analyzed by XPS, while the supernatant was filtered (0.22 μm PTFE syringe filter) and analyzed by ICP-MS. The ICP analysis revealed the release of Na^+^ and Yb^3+^ ions as well as a *ca*. 16% of Au^3+^ cations with respect to the added Au^3+^ quantity, thereby emphasizing the occurrence of Au adsorption on the LnNP surface/insertion in the LnNP. Moreover, XPS can reveal the ratio of the different oxidation states of gold by deconvoluting the Au 4f core-level photoemission spectrum corresponding to Au 4f_7/2_ and 4f_5/2_ (binding energies: 84.2 and 87.9 eV for Au^0^_;_ 85.0 and 88.7 eV for Au^+^; and 87.0 and 90.9 eV for Au^3+^).^41^ In this way, Au 4f XPS of the pellet revealed the presence of Au species in the LnNP (Figure S3a, Table S1). The main Au species was Au^0^ due to the fact that DMF acted as a mild reducing agent. This result indicates that Au species are adsorbed on/inserted in the LnNP and confirmed the formation of NHS. In this way, we hypothesize that Au^+^ and Au^3+^ cations can assemble in the surface through ionic interaction, and some exchange of Na^+^ and Ln^3+^ (Y^3+^, Yb^3+^, and Tm^3+^) occurs on LnNP surface positions; thanks to the similar size of the cations,^42^ thereby creating Au nanodomains inserted within the LnNP surface. Note that, even though three different gold species are detected by XPS, at this point, AuNCs have not been formed yet, as confirmed by attenuance and PL spectra (Figure S4a).

After pretreatment of the UC_Tm_@BF_4_ dispersion with Au^3+^, an excess of GSH was added to the mixture, which was then stirred for 1 h. Next, the reaction mixture was left in the dark without stirring or shaking in a closed microcentrifuge tube. The evolution of the dispersion was followed by XPS, TEM, PL, and attenuance. XPS analysis was performed for the solid pellet obtained after centrifuging the reaction mixture. Immediately after the addition of GSH, the dispersion turned dark brown, indicating the formation of AuNCs,^43^ but very quickly (in less than 2 min), the dispersion became colorless. As expected, GSH efficiently reduced most Au^3+^ ions to Au^+^ ions, as evidenced by the drastic decrease of Au^3+^ absorption after several days (Figure S4) and the predominance of Au^+^ (Figure S3). TEM images showed the creation of an amorphous layer of GSH containing the generated AuNCs. AuNCs were also observed in the dispersion (i.e., not in the proximity of LnNP). From a few minutes after the addition of GSH until 24 h later, the AuNCs grew on the LnNP surface as shown in the TEM images (Figure S5), in which high-contrast spotted dots around LnNPs and large isolated AuNC aggregates (high-contrast areas without LnNPs) were observed. The formation of AuNCs was corroborated by their PL: two emission bands centered at 420 and 750 nm (Figure S5a), thus indicating the formation of different-sized AuNCs. Gradually, the thermodynamic equilibrium between the free large AuNC aggregates and the AuNCs-capped LnNPs moved forward and produced UC_Tm_@AuNC NHS after 48–72h, while the AuNC aggregates disappeared in a size-focusing manner.^11^ From this point, changes in the cluster morphology/size started to occur: the emission became purer (only one band) and it shifted to shorter wavelengths (Figure S4a), thereby indicating a relative monodisperse size population of the AuNCs; the ratio Au^0^/Au^+^ kept growing (Table S1) and the Au^3+^ ratio decreased while the total gold content in the sample increased (from 3% right after adding GSH to 40% 1 week later, data from XPS). This is consistent with the existence of gold species either adsorbed or included in the UC_Tm_ core presumably by cation exchange.^38^ Moreover, 6 days after GSH addition, the absorption band of Au^3+^ disappeared and the shape of AuNC absorption could be glimpsed in the crude (Figure S4b). Finally, 7 days after GSH addition, a maximum of emission was observed (even by the naked eye under UV lamp), and the pale-brown dispersion was purified by centrifugation-redispersion washes (H_2_O:ACN, 1:3; 18,000 *g*, 15 min). After that, the UC_Tm_@AuNC NHS was dispersed in D_2_O, for optical characterization, or H_2_O for cell viability assays.

Therefore, this easy and user-friendly synthetic protocol can be seen as a cation exchange pretreatment (1h) of the LnNP with Au^3+^, followed by a mild reduction and an aging period (7 days) with GSH. Note that control experiments were also performed: (i) addition of Au^3+^ and GSH immediately (skipping the 1-h Au^3+^ pretreatment) and (ii) mixing of the preformed AuNC and LnNP (under otherwise identical experimental conditions); none of them succeeded in preparing the UC_Tm_@AuNC NHS. These control experiments proved that Au^3+^ cationic seeding was crucial to grow the AuNC onto the LnNP surface.

For comparative purposes, AuNCs (average size of 2.0 ± 0.4 nm) were prepared by following the same methodology to that used in the preparation of the UC_Tm_@AuNCs (see materials and methods and Figure S6). Additionally, comparable LnNPs were prepared with the same UC_Tm_@BF_4_ batch followed by GSH functionalization and purification (termed UC_Tm_ for simplicity).

### Morphological Characterization of the UC_Tm_@AuNC NHS

The as-prepared UC_Tm_@AuNC NHS shows a uniform raspberry-like morphology. An organic shell around the UC_Tm_ core can be distinguished in which homogeneously distributed AuNCs (1.5 ± 0.5 nm) are the drupelets around the UC_Tm_ receptacle (no AuNCs were observed in the representative areas explored on the grid). The size distribution of the core LnNP (length × width of 27.1 ± 2.5 × 23.3 ± 2.2 nm) proved that there were no significant changes other than the coating with the organic shell and the presence of AuNCs on their surface (Figure 1b,c). The presence of Au in the sample was also corroborated by energy dispersive X-ray spectrometry (EDAX) (Figure S7). An average of 32 AuNC per UC_Tm_ was estimated from the high-contrast dark dots per LnNPs of the HRTEM images. Elemental mapping images of UC_Tm_@AuNC NHS show that Au, Yb, and Y elements are uniformly distributed (Figure 1d–h).

The weight contribution of GSH (ca. 45%) in the UC_Tm_@AuNC NHS was determined by TGA and compared with UC_Tm_ and AuNC. In all cases (UC_Tm_@AuNC NHS, UC_Tm_, and AuNC), the typical weight loss of GSH (at 192 °C)^44^ occurred at 221 °C, suggesting a higher thermal stability of the GSH when attached to the nanomaterial surface. In UC_Tm_, the weight contribution of GSH was only around 6 wt %, whereas in the AuNC, it was 33% (Figure S8). After an initial loss of water (4%), three characteristic steps attributing to GSH were distinguished and perfectly matched those reported for GSH-capped Pt clusters.^44^ The first one (200–300 °C) amounts to 10%, the second one (300–450 °C) to 15%, and the third one from 450 to 950 °C (16%).

XPS studies confirmed the existence of Au in the different oxidation states Au^0^, Au^+^, and Au^3+^ for UC_Tm_@AuNC (86.0, 10.3, and 3.7%, respectively; Figure S3d, Table S1) and confirmed different amounts of gold species for the AuNC sample: 80.5% of Au^0^ and 19.5% of Au^+^ (Figure S9 and Table S1). The S 2p peaks were found in the 167–157 eV range for UC_Tm_@AuNC NHS (Figure S10). The peaks could be decomposed into several peaks, with some of them being attributed to S 2p centered at 162.9, 164.5, and 165.6 eV, ascribed to S–Au, S–C and S–S bonds, respectively.^45^ The absence of S–H signals was indicative of glutathione in the thiolate form, and the smallest area of the S–S signal was consistent with some dimerization of GSH to glutathione disulfide (GSSG); the oxidation of GSH to GSSG is proven useful to coat AuNCs.^45^

### Photophysical Properties of the UC_Tm_@AuNC NHS

**Figure 2:**
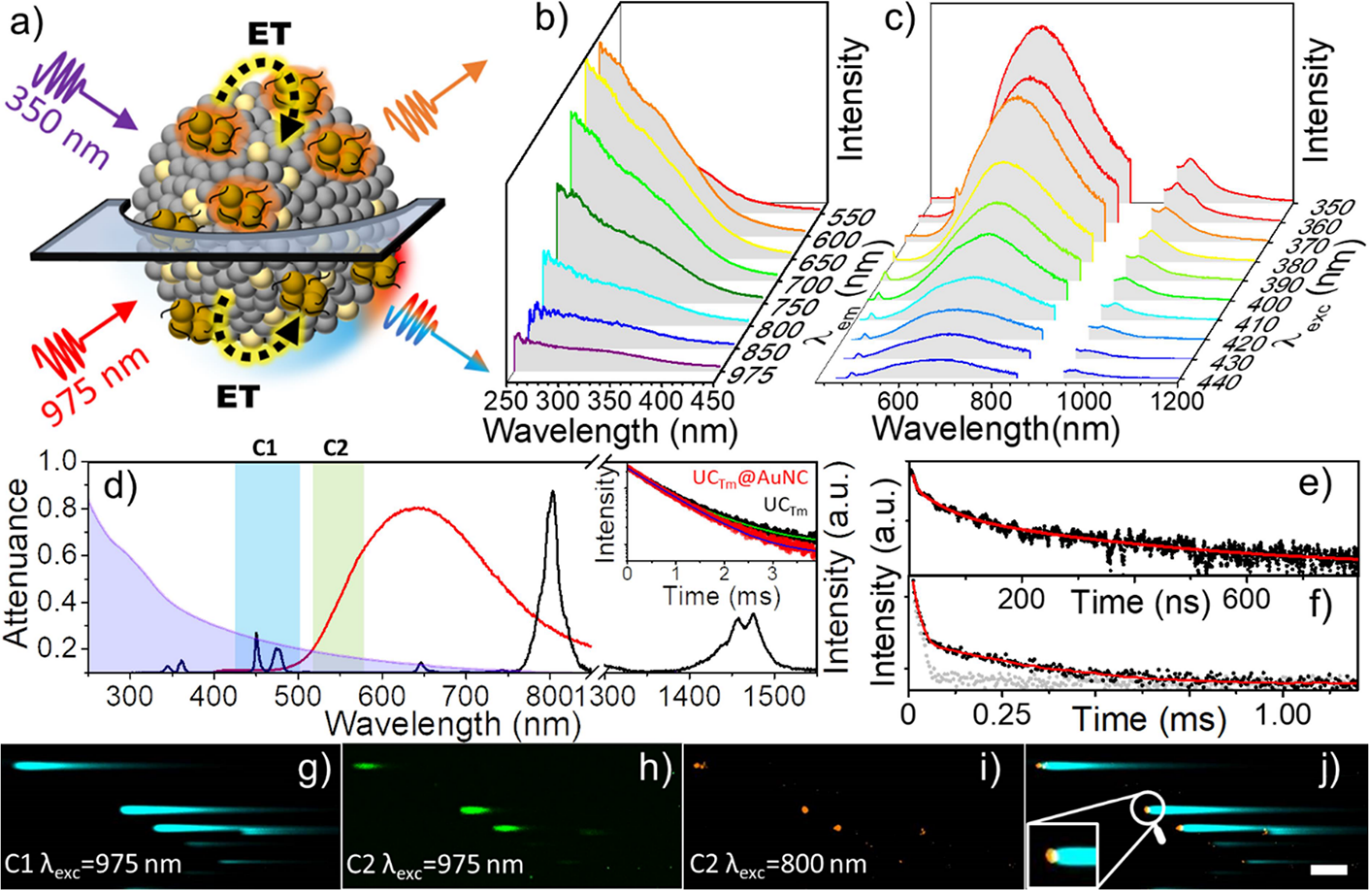
Photophysical characterization of UC_Tm_@AuNC NHS in D_2_O. (a) Scheme of the dual emission modes exhibited by the UC_Tm_@AuNC under 350 and 975 nm excitation; ET states for energy transfer. (b) 0.5 mg/mL UC_Tm_@AuNC excitation map at the indicated emission wavelengths. The excitation spectrum registered at 975 nm has been amplified x5 times to visualize the trend. (c) 0.5 mg/mL UC_Tm_@AuNC emission map at the indicated excitation wavelengths. (d) Attenuance (colored area), emission spectrum (λ_exc_ 350 nm; red line), and upconversion and downshifting emission spectrum (λ_exc_ 975 nm; PD 9 W·cm^–2^; black line) of 0.5 mg/mL UC_Tm_@AuNC. Colored rectangles show the two detection channels of the NIR-LSM technique. Inset: kinetic profiles (λ_em_ 800 nm) of UC_Tm_@AuNC (red dots) and UC_Tm_ (black dots). Kinetic profile and fitting of the UC_Tm_@AuNC at (e) 600 nm (λ_exc_ 355 nm) and (f) 1000 nm (λ_exc_350 nm) where the IRF is indicated in gray. (g–j) NIR-LSM images of the same area of the UC_Tm_@AuNC sample. (g) Tm^3+^ upconversion emission (λ_exc_ 975 nm; dwell time: 8 μs·pixel^–1^; F 59 J·cm^–2^). (h) Lifetime lengthening of the AuNC (λ_exc_ 975 nm; dwell time: 8 μs·pixel^–1^; F 59 J·cm^–2^). (i) Emission of AuNCs (two-photon excitation; λ_exc_ 800 nm; dwell time: 4 μs·pixel^–1^; F 27 J·cm^–2^). (j) Merging of panels g and i. Scale bar of 25 μm applies for images g–i.

Figure 2 shows the photophysical characterization of UC_Tm_@AuNC NHS in D_2_O. Figure 2a illustrates the dual emission modes exhibited by UC_Tm_@AuNC under 350 and 975 nm excitation. Although it will not be discussed in this article, the characterization in H_2_O is shown in the Supporting Information (Figure S11) and displayed a similar spectral attenuance and emission shape but slightly worse PL performance (vide infra). Control samples, such as AuNC and UC_Tm_, were also dispersed in D_2_O.

Figure 2d shows the attenuance spectrum of UC_Tm_@AuNC NHS with the characteristic broad absorption band of AuNC together with the scattering due to UC_Tm_. The shape of the AuNC in the NHS is very similar to that of the control AuNCs, showing an absorption band at ca. 320 nm.

Excitation–emission maps (λ_exc_ 350–440 and 980 nm; λ_em_ 550–850 nm) were acquired to obtain a complete spectral fingerprint of the AuNC present in the UC_Tm_@AuNC NHS (Figure 2b,c). The excitation spectra revealed a shoulder at 350 nm, that is, where the AuNCs absorb, whereas the emission spectra showed two distinctive emissions. The most intense emissions were observed upon excitation of UC_Tm_@AuNC at 350 nm. One of them is a multicolor emission due to the characteristic AuNC emission from 500 to 900 nm (λ_em,max_ shifting from 600 to 700 nm as the excitation wavelength varies from 350 to 440 nm). Such large Stokes shifts (ca. 250 nm) are common for AuNCs. The other emission band at longer wavelengths (maximum emission wavelength λ_em,max_ at 978 nm, λ_exc_ at 350 nm) corresponds to Yb^3+^ (^2^F_5/2_ → ^2^F_7/2_) (Figure 2c).

Note that the excitation spectrum registered at 978 nm revealed a broad band with a shoulder at 350 nm (Figure S12), and PL control experiments (λ_exc_ 350 nm, Figure S13) proved that the AuNC control sample did not display Yb emission in the NIR. This is indicative of energy transfer from AuNCs to Yb^3+^ within the NHS. Up to our knowledge, this is the first example of Yb sensitization by AuNCs.

The kinetic profiles of UC_Tm_@AuNC NHS, upon excitation at 355 nm, were recorded at 600 nm (see Table 1 and Figure 2e) and 1000 nm (Figure 2f). The kinetic decays at 600 nm fitted to a triexponential function and showed a similar PL average lifetime (τ_PL,av_) to that of the AuNC in the UC_Tm_@AuNC NHS as compared with that of AuNCs (201 ns vs 202 ns, respectively). Both display the instrument response function (IRF) relaxation (4 ns) as well as a fast component (40 and 30 ns for AuNC in the UC_Tm_@AuNC NHS and AuNC, respectively) and a slower one (260 and 250 ns for AuNCs in the UC_Tm_@AuNC NHS and AuNCs, respectively). These decay components have been attributed to metal–metal transition and ligand-to-metal charge transfer, respectively.^43^ The equal behavior observed in the kinetics of AuNC PL discards the occurrence of RET to Yb^3+^. Therefore, the sensitized emission of Yb^3+^ must come from a trivial energy transfer directly from the AuNC (NIR photons of AuNC → Yb^3+^) or through Tm^3+^ ions (vis-NIR photons of AuNC → Tm^3+^ → Yb^3+^).

**Table 1:** AuNC and UC_Tm_@AuNC NHS Photoluminescence Average Lifetimes (τ_PL,av_) (0.5 mg/mL in D_2_O), Photoluminescence Lifetime Components (τ_PL,n_), and Its Contribution to the Decay (*A*_n_) Recorded at 600 nm upon 355 nm Excitation

sample	τ_PL,av_ (ns)	τ_PL,1_ (ns)	*A*_1_ (%)	τ_PL,2_ (ns)	*A*_2_ (%)	τ_PL,3_ (ns)	*A*_3_ (%)
AuNC	202.3	3.8 ± 0.1	1.82	28.7 ± 0.4	18.79	247.9 ± 3.3	79.39
UC_Tm_@AuNC	200.9	4.1 ± 0.1	2.57	42.3 ± 0.5	25.15	263.1 ± 4.4	72.28

The attenuance and emission spectra together with the emission kinetics at 600 nm seems to fit well to the formation of small GSH capped-AuNCs of the formula Au_10–12_GSH_10–12_^43,46,47^ on the LnNP surface. However, we do not discard the presence of larger species due to the emission tail observed at long wavelengths.

The antenna effect can be observed for UC_Tm_@AuNC NHS in deuterated water, upon 350 nm excitation, thus resulting in long-lived Yb excited-state lifetime (μs) as previously observed for organic dye-sensitized LnNPs^48,49^ and AuNPs with pendant ytterbium(III) ions.^50^ The emission of Tm^3+^ was not detected probably due to the negligible overlapping between the AuNC emission and Tm absorption or to the low Tm concentration (<0.3%) in the UC_Tm_ used to construct the NHS.

The kinetic profiles of UC_Tm_ and UC_Tm_@AuNC NHS at 1000 nm, attributing to Yb^3+^ emission upon excitation at 350 nm, were also recorded. The kinetic decays fitted to a monoexponential function. The lifetime of the Yb^3+^-sensitized emission in the UC_Tm_@AuNC was longer than that from direct excitation of Yb^3+^ in UC_Tm_ (228 ± 7 μs vs 154 ± 2 μs, Figure 2f vs Figure S13b), highlighting the passivating effect of AuNCs in lanthanide emission, which reduces deactivation processes of Yb^3+^.

The Φ_PL_ of AuNCs in the UC_Tm_@AuNC NHS in D_2_O was 3.6%, while the control AuNC sample afforded 1.9%. The Φ_PL_ enhancement can be attributed to (i) the AuNC insertion in the NHS, hence restricting the intermolecular vibrations, relaxation, and rotations of the Au(I)-GSH^39^ and/or (ii) the contribution of the NIR emission upon Yb sensitization. These values correlate well with emission quantum yields ranging from 4.1 to 1.8%, previously reported for the AuNC^45^ and the emission enhancement reported for Sm^3+^, Y^3+^, and Yb^3+^-coated AuNCs^39^ and adenosine monophosphate-capped AuNCs treated with Yb.^51^

NIR excitation of UC_Tm_@AuNC (λ_exc_ 980 nm), where UC_Tm_ absorbs, showed the characteristic Tm^3+^ emission bands of the upconverting core,^52^ corresponding to ^3^P_6_ → ^3^F_4_ (345 nm), ^1^D_2_ → ^3^H_6_ (368 nm), ^1^D_2_ → ^3^F_4_ (450 nm), ^1^G_4_ → ^3^H_6_ (475 nm), ^1^G_4_ → ^3^F_4_, (644 nm), and ^3^H_4_ → ^3^H_6_ (800 nm) together with that of ^2^F_5/2_ → ^2^F_7/2_ (1000 nm) and ^3^H_4_ → ^3^F_4_ (1475 nm) due to the downshifting emission of Yb^3+^ and Tm^3+^, respectively (Figures S14 and S15 for the comparison with the upconversion emission spectrum of UC_Tm_). The upconversion quantum yield (UCQY) for thw UC_Tm_@AuNC solid was estimated as (3.0 ± 0.3)·10^–4^, that is, lower than that of UC_Tm_ dispersion (5.0 ± 0.5)·10^–3^ (Figure S16). Moreover, the presence of gold species in the UC_Tm_ dispersion after 1h of cation exchange with Au^3+^ and purification translated into an enhancement of the UCQY(7.0 ± 0.5)·10^–3^. Therefore, the UCQY decrease can be attributed mainly to the presence of AuNCs, which decreases the efficiency of the upconversion process. Additionally, this passivation can be also observed in the kinetics of the Yb^3+^ downshifting emission (λ_exc_ 980 nm, λ_em_ 1000 nm): its lifetime was lengthened x2.5 times (from 78 to 199 μs for UC_Tm_ and UC_Tm_@AuNC, respectively; see Figure S17 and Table S3).

Interestingly, even in the cation exchange step (before GSH addition), gold species adsorbed on and/or inserted in the LnNP produced a x2 times lifetime lengthening (see Figure S17 and Table S3).

The upconversion emission lifetimes (λ_exc_ 980 nm) of UC_Tm_@AuNC were influenced by the presence of AuNCs (note that their absorption overlaps with the whole emission spectrum of the UC_Tm_ core; see Figure 2d). The shorter emission lifetimes calculated for the ^1^G_4_ → ^3^H_6_, ^1^G_4_ → ^3^F_4_, and ^3^H_4_ → ^3^H_6_ transitions of the UC_Tm_@AuNC NHS, as compared to those of UC_Tm_, suggest a RET process from the UC_Tm_ to the AuNC present on the NHS (Table 2); however, RET cannot be asserted firmly only by the lifetime shortening of the upconversion emissions,^53^ especially using long pulse excitation sources.^54^ A similar result has been previously reported for multilayer LnNPs (dopped with Yb, Er) covalently linked to Au_25_.^18^ Although some publications report the ^1^O_2_ sensitization from AuNCs,^33,35^ its characteristic phosphorescence at 1260 nm was neither detected under our experimental conditions at 350 nm (Xe Lamp) nor at 980 nm excitation (PD of 9 W·cm^–2^).

**Table 2:** Tm^3+^ PL Lifetimes of of UC_Tm_ and UC_Tm_@AuNC (0.5 mg/mL) Recorded upon 980 nm Laser Excitation

	sample	τ_av_ (μs)	τ_1_ (μs)/*A*_1_(%)	τ_2_ (μs)/*A*_2_ (%)	χ^2^
^1^D_2_ → ^3^H_6_ (360 nm)	UC_Tm_	134.6 ± 1.2			1.0079
	UC_Tm_@AuNC	213.5 ± 0.9			1.3133
^1^D_2_ → ^3^F_4_ (450 nm)	UC_Tm_	151.4	118.6 ± 2.8/84.5	330.6 ± 47.6/15.5	1.0232
	UC_Tm_@AuNC	196.5 ± 0.7			1.1156
^1^G_4_ → ^3^H_6_ (475 nm)	UC_Tm_	628.4	370.1 ± 22.1/20.6	695.5 ± 11.3/79.4	1.0300
	UC_Tm_@AuNC	570.5 ± 1.0			1.3079
^1^G_4_ → ^3^F_4_ (644 nm)	UC_Tm_	624.0	300.3 ± 27.1/13.8	675.7 ± 13.0/86.2	0.9817
	UC_Tm_@AuNC	562.1 ± 1.0			1.3317
^3^H_4_ → ^3^H_6_ (800 nm)	UC_Tm_	779.2	477.4 ± 11.6/43.3	1009.3 ± 24.2/56.8	1.0595
	UC_Tm_@AuNC	701.7 ± 2.1			1.5104
^3^H_4_ → ^3^F_4_ (1470 nm)	UC_Tm_	687.3 ± 5.5			1.2655
	UC_Tm_@AuNC	689.3 ± 3.5			1.2976

An enhancement in the upconversion luminescence lifetime for both ^1^D_2_ → ^3^H_6_ (368 nm), ^1^D_2_ → ^3^F_4_ (450 nm) can be observed; this is indicative of a longer time of the ^1^D_2_ level remaining in the system. This is probably due to a passivating effect of the AuNC in the UC_Tm_@AuNC NHS surface from deactivation processes that translate into a decrease in the nonradiative relaxation from ^3^F_4_ to ^3^H_4_ followed by the excited state absorption ^3^H_4_ → ^1^D_2_.^55^

The AuNC sensitized emission was not detected under these experimental conditions (regular fluorometer) probably due to the low emission (Φ_PL_ 3.6%) and the low excitation power at 980 nm. Near infrared laser scanning microscopy technique (NIR-LSM) was then used to further confirm the RET process and homogeneity of the UC_Tm_@AuNC sample (i.e., although each NHS has heterogeneous composition due to the presence of UC_Tm_ core and the AuNC in each NHS, all of them displayed a similar optical response; Figure S18).^35,56^Figure 2g shows an image of UC_Tm_@AuNC showing homogeneously distributed long emission tails acquired between 420 and 500 nm (λ_exc_ 980 nm; 8 μs·pixel^–1^), which can be attributed to Tm^3+^ (τ_PL,av_ 494 μs).^35^ The emission detected in NIR-LSM is a mixture of 450 nm (^1^D_2_ → ^3^F_4_) and 475 nm (^1^G_4_ → ^3^H_6_) Tm^3+^ upconversion emission bands due to the detection channel spectral range (C1:420–500 nm); therefore, it must reflect this dual character. In fact, the kinetics fit a biexponential decay reflecting both transitions and, in addition, the lifetimes show the identical behavior observed in the fluorometer (see Table 2): lengthening of the first relatively short component attributed to 450 nm transition, and shortening of the second long component attributed to the 475 nm transition (Figure S19 and Table S4).

The RET efficiencies are hard to quantify in LnNPs as energy is stored in the Yb sensitizers during RET from activators to RET acceptors.^54,57^ Thus, the shortening of the 450 nm-attributed component is not a good indicator of RET from Yb to Tm and the intensity changes cannot be evaluated under our experimental conditions.

Moreover, an emission tail was observed for UC_Tm_@AuNC in C2 (λ_em_ 515–580 nm; λ_exc_ 980 nm), while no emission was observed for UC_Tm_ in the same detection channel under identical conditions (Figure S18). This emission tail (Figure 2h) can be attributed to the lifetime lengthening (124 μs) of AuNC in the UC_Tm_@AuNC NHS which reflects the Tm^3+^ precursor lifetime and proves the RET.^58−60^ An emissive spot is detected (no emission tail) upon two-photon excitation of AuNCs in the UC_Tm_@AuNC at λ_exc_ 800 nm (4 μs·pixel^–1^) in the same emission channel (C2), where the UC_Tm_ core does not absorb (Figure 2i). This spot reflects the shorter lifetime of AuNCs upon two-photon excitation (λ_exc_ 800 nm) as compared to the RET process (λ_exc_ = 980 nm) (see Figure S20 for comparison with AuNCs and UC_Tm_ at λ_exc_ 800 nm). The visual colocalization of the AuNC emission upon two-photon excitation and the Tm^3+^ upconversion emission is clearly demonstrated by overlapping Figure 2g,i images (Figure 2j).

### Cytotoxicity Study

Since UC_Tm_@AuNC NHSs are dispersible in water, they could be used as biological probes. In the present work, the viability of UC_Tm_@AuNC and the corresponding counterparts as controls (UC_Tm_ and AuNC) were evaluated in vitro on HeLa human primary cell lines.

HeLa cells were seeded for 24 h. Then, the culture medium was removed, and cells were treated with concentrations ranging from 0 to of 800 μg·mL^–1^ for 24 h of incubation. Cell viability was evaluated through a standard MTT assay. Nontreated cells and cells exposed to positive controls (H_2_O_2_ and tertbutyl hydroperoxide) were used as a reference for cell viability. HeLa cells exhibited a cell viability higher than 95% even at 500 μg·mL^–1^ of UC_Tm_@AuNC (81% at 800 μg·mL^–1^), whereas cell viability was slightly reduced for UC_Tm_ (86%) and AuNCs (87%) at 500 μg·mL^–1^. In any case and according to the definition of cytotoxicity in the ISO10993-5 guideline for medical devices,^61^ none of them show any indication for cytotoxicity in HeLa cells (Figure S21) since cell viability was higher than 70% for all the tested concentrations. A negative control was also performed by exposing cells to a culture medium with water at the same percentage of the samples.

**Figure 3:**
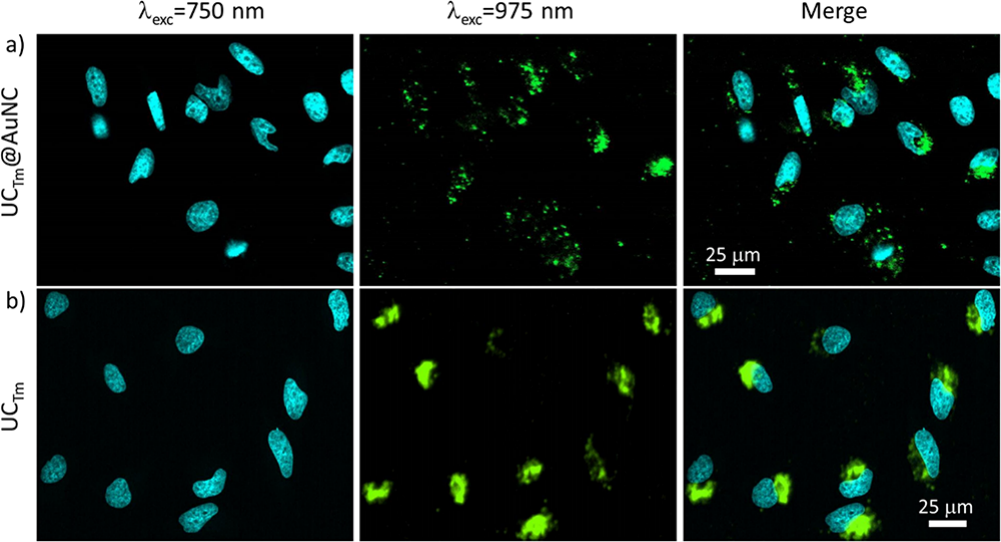
NIR-LSM images of HeLa cells stained with Hoechst 33342 and exposed to (a) UC_Tm_@AuNC and (b) UC_Tm_ for 24 h at 37 °C (λ_em_ 420–500 nm applies for all images). (Left) Fluorescence signal of Hoechst 33342 (λ_exc_ 750 nm; dwell time: 4 μs·pixel^–1^). (Middle) Tm^3+^ upconversion emission (λ_exc_ 975 nm; dwell time: 100 μs·pixel^–1^). (Right) Composite of the in line previous images. 25 μm scale bar applies for all images. The intensity of the images has been magnified for visualization purposes.

Preliminary studies were also carried out to evaluate the capabilities of the UC_Tm_@AuNC NHS for imaging upon NIR excitation as previously described for UC_Tm_@BF_4_.^62^ The concentration used for the imaging experiments in HeLa cells was 200 μg·mL^–1^. Fixed cell imaging experiments were conducted for UC_Tm_@AuNC and UC_Tm_. The nuclear staining with Hoechst 33342 enables their excitation through two-photon absorption at 750 nm (Figure 3, left). The emission microscopy images of UC_Tm_@AuNC inside the cells are shown in Figure 3a (middle) (λ_exc_ 975 nm) in the same area imaged previously with Hoechst. UC_Tm_ is also shown for comparison in Figure 3b (middle). Long dwell time (slow scanning speed) was used to avoid the characteristic tail of the long-lived lanthanide emitters (Figure 2g), which spread its luminescence several pixels away in the scanning direction if the scanning speed is not slow enough.^35^ The overlapping of previous images of the same area clearly showed the cellular internalization of UC_Tm_@AuNC and UC_Tm_ in HeLa cancer cell lines. The emission due to AuNCs in the cell was not detected under our experimental conditions neither after two-photon excitation (λ_exc_ 800 or 750 nm) nor RET (λ_exc_ 975 nm).

## Conclusions

In summary, we have developed a novel, successful strategy to easily synthesize a colloidal raspberry-like UC_Tm_@AuNC NHS by using bare UC_Tm_ as the template for gold cation exchange to generate the desired seed for AuNC growth in situ. Then, GSH was used to coat the cations on the nanoparticle surface while directing the reduction of Au^3+^ to Au^0^ and Au^+^ in a subsequent diffusion aging growth step, thus simplifying the synthesis of the LnNP@AuNC NHS.

The emission due to Yb sensitization and that of the AuNC were registered upon excitation at 350 nm, whereas upon NIR-excitation (980 nm) of the NHS, the upconverting emission of UC_Tm_, the down-shifting emission of thulium, and multicolored, long-lived emissions of both the UC_Tm_ and AuNC can be observed together with the two-photon emission of the AuNC, which occurs in the nanosecond scale.

Moreover, the UC_Tm_@AuNC NHS showed good biocompatibility, was taken up by HeLa cells, and was used for bioimaging upon NIR excitation.

Future studies using this simple synthetic methodology will be carried out to explore other lanthanide doping (e.g., Er^3+^_,_ Nd^3+^) or other LnNP inorganic matrices to optimize the aging period under different conditions (T, P, ...), to test other thiolate ligands (e.g., disulfide), and to drive the AuNCs size in the aging period to tune the absorption and emission wavelengths of each counterpart to expand their imaging capabilities. These variations will result in new optical properties of the nanoheterostructure.

## Supporting Information Available

Experimental procedure for the synthesis of UC_Tm_@OA and UC_Tm_@BF_4_; XRD pattern and TEM of UC_Tm_@OA; FTIR spectra of UC_Tm_@OA and UC_Tm_@BF_4_; XPS spectra of UC_Tm_@AuNC and AuNC before and after cation exchange; TEM images of the evolution of the UC_Tm_@AuNC synthesis; absorption and emission spectra of the UC_Tm_@AuNC synthesis; characterization of AuNCs. EDAX of UC_Tm_@AuNC; TGA analyses of UC_Tm_, UC_Tm_@AuNC, AuNC, and GSH; Au 4f XPS spectrum of AuNCs; S 2p XPS spectrum of UC_Tm_@AuNC; photophysical characterization of UC_Tm_@AuNC in H_2_O; excitation spectra of UC_Tm_ and UC_Tm_@AuNC (λ_em_ 978 nm); emission spectra (λ_exc_ 350 nm) of UC_Tm_@AuNC and UC_Tm_; energy level diagram of NaYF_4_:Yb^3+^, Tm^3+^ LnNP; emission spectra (λ_exc_ 980 nm) of UC_Tm_, UCQY; kinetic profiles (λ_exc_ 975 nm, λ_em_ 1000 nm); NIR-LSM images of UC_Tm_ and UC_Tm_@AuNC at λ_exc_ 975 nm; kinetics obtained from NIR-LSM (λ_exc_ 975 nm); NIR-LSM images of UC_Tm_ and AuNC at λ_exc_ 800 nm; and in vitro cell viability (DOCX)
