# Supplementary material for: Raspberry-like Nanoheterostructures Comprising Glutathione-Capped Gold Nanoclusters Grown on the Lanthanide Nanoparticle Surface
Source: Chem Mater. 2024 Mar 5;36(9):4426–36. doi: 10.1021/acs.chemmater.3c03333 (PMC11099914; doi:10.1021/acs.chemmater.3c03333)
Supplement: Supplementary file 1 — cm3c03333_si_001.docx [file cm3c03333_si_001.docx]

Raspberry-like nanoheterostructures comprising glutathione-capped gold nanoclusters grown on the lanthanide nanoparticle surface

Irene Pérez-Herráez^a,‡^, Juan Ferrera-González,^a,‡^, Elena Zaballos-García^b^, María González-Béjar^a,*^, Julia Pérez-Prieto^a,*^

^a^Instituto de Ciencia Molecular (ICMol), Departamento de Química Orgánica, Universitat de València, C/ Catedrático José Beltrán, 2, Paterna, Valencia 46980, Spain.

^b^Department of Organic Chemistry, Universitat de València, Av. Vicent Andrés Estellés s/n, 46100, Burjassot, Valencia, Spain

**Index**

Synthesis of UC_Tm_@OA 3

Synthesis of UC_Tm_@BF_4_ 4

**Figure S1**. XRD pattern and TEM image of UC_Tm_@OA 5

**Figure S2**. FTIR spectra of UC_Tm_@OA and UC_Tm_@BF_4_ 6

**Figure S3.** Au4f XPS spectra of the UC_Tm_@AuNC synthesis 7

**Table S1.** XPS ratio of Au^0^, Au^+^ y Au^3+^ of UC_Tm_@AuNC and AuNC 7

**Figure S4.** Absorption and emission spectra of UC_Tm_@AuNC synthesis 8

**Figure S5.** TEM images of the UC_Tm_@AuNC synthesis 9

**Figure S6**. Characterization of AuNC 10

**Figure S7**. EDAX spectrum of UC_Tm_@AuNC 11

**Figure S8**. TGA of UC_Tm_@AuNC, UC_Tm_ and AuNC 12

**Figure S9**. Au4f XPS spectrum of AuNC 13

**Figure S10**. S2p XPS spectrum of UC_Tm_@AuNC 13

**Figure S11**. Photophysical characterization of UC_Tm_@AuNC in H_2_O 14

**Figure S12.** Excitation spectra of UC_Tm_ and UC_Tm_@AuNC (λ_em_ 978 nm) 15

**Figure S13**. Emission spectra (λ_exc_ 350 nm) of UC_Tm_@AuNC and UC_Tm_ 16

**Figure S14**. Energy level diagram of NaYF_4_:Yb^3+^, Tm^3+^ UCNP 17

**Figure S15**. Emission spectra (λ_exc_ 980 nm) of UC_Tm_ 17

**Figure S16**. UCQY 18

**Figure S17.** Kinetic profiles (λ_exc_ 975 nm, λ_em_ 1000 nm) 18

**Table S2.** Yb^3+^ PL lifetimes (λ_exc_ 980 nm). 19

**Figure S18**. NIR-LSM images of UC_Tm_ and UC_Tm_@AuNC (λ_exc_ 975 nm) 19

**Figure S19**. Kinetics obtained from NIR-LSM 20

**Table S3.** Fitting parameters of NIR-LSM kinetics 20

**Figure S20**. NIR-LSM images of UC_Tm_ and AuNC (λ_exc_ 800 nm) 21

**Figure S21**. Cell viability 22

References 22

**Synthesis of UC_Tm_@OA.** The oleate-capped β-NaYF4:Yb^3+^(24.9 %),Tm^3+^(0.3 %) LnNPs were synthesized by thermal decomposition with oleic acid and octadecene at high temperature following a well-known protocol.^1–7^ Briefly, oleic acid (160 mL) and 1-octadecene (300 mL) were added to a 1 L three-necked round bottom flask. Then a lanthanide mixture, containing YCl_3_·6H_2_O (15 mmol), YbCl_3_·6H_2_O (5 mmol) and TmCl_3_·6H_2_O (0.06 mmol), in methanol (25 mL) was added to the flask. The mixture was stirred severely and kept under N_2_ atmosphere until the end of the synthesis. The mixture was heated to 160 ºC until the complete dissolution of the lanthanide salts. The solution was then allowed to cool down to room temperature and 60 mL of a methanol solution containing NaOH (50 mmol) and NH_4_F (80 mmol) was added at once to the flask. The mixture was stirred for 30 minutes at 60 ºC and then heated to 305 ºC for 90 minutes. Afterwards, the mixture was allowed to cool to room temperature. The formed LnNPs were precipitated by the addition of ethanol and isolated by centrifugation (13000 g for 6 minutes). The white pellet was washed five times by redispersion-centrifugation cycles. The washes consisted of redispersing the LnNP pellet in a “good solvent” (apolar solvent), then add an excess of a “bad solvent” (polar) to induce the precipitation of the UCNP and eventually centrifuging the dispersion (13000 g for 6 minutes). Firstly, two washes were performed with chloroform and ethanol and next three washes were done with cyclohexane and acetone. Finally, the purified UC_Tm_@OA were redispersed in cyclohexane and a weak centrifugation (1000 g for 3 minutes) was carried out to discard the aggregates (precipitate). The concentration (mg·mL^-1^) of the UC_Tm_@OA in the supernatant was obtained by difference in weight by drying an aliquot of the dispersion.

**Synthesis of UC_Tm_@BF_4_**. A ligand-exchange strategy with nitrosonium tetrafluoroborate (NOBF_4_) was carried out to replace the original oleate ligands attached to the LnNPs.^8,9^ Briefly, 22.5 mL of UC_Tm_@OA dispersed in cyclohexane (44.5 mg·mL^-1^) and 12.5 mL of DMF were mixed in a round flask. This resulted in a two-phase system consisting of an upper layer of cyclohexane (UC_Tm_@OA) and a subjacent layer of DMF. Subsequently, nearly the same mass of UCNP is added of NOBF_4_ (1075 mg) at once. The biphasic mixture was maintained under vigorous stirring for 3 h at *ca.* 30 ºC. This resulted in the phase transfer of the UCNP from cyclohexane to DMF. The upconverting material in the slightly turbid DMF phase was precipitated by adding chloroform and collected by centrifugation (6000 g for 6 minutes). The transparent pellet was washed three times by redispersion in DMF and then reprecipitation by addition of an excess of chloroform and centrifugation (6000 g for 6 minutes). Finally, the purified UC_Tm_@BF_4_ were dispersed in DMF and a weak centrifugation (1000 g for 3 minutes) was performed to discard the aggregates (precipitate). The concentration (mg·mL^-1^) of the UC_Tm_@BF_4_ in the supernatant was obtained by difference in weight by drying an aliquot of the dispersion.


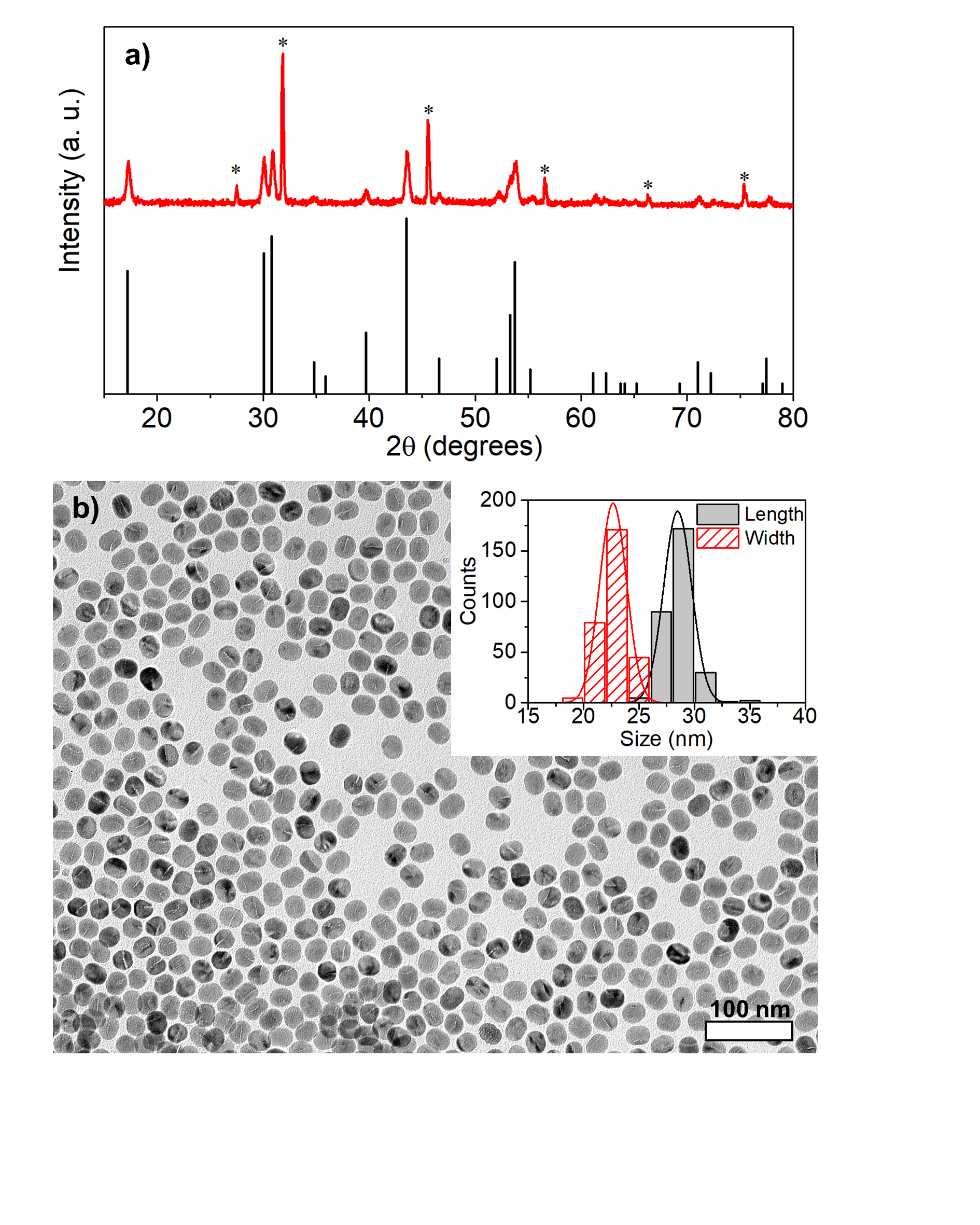


**Figure S1**. (a) XRD pattern of hexagonal prims of β-UC_Tm_@OA (red line) and the reference: β‑NaYF_4_ (PDF 16-0334) (black). NaCl signals (*). (b) TEM image and size distribution of UC_Tm_@OA.





**Figure S2**. FTIR spectra of UC_Tm_@OA (red line) and UC_Tm_@BF_4_ (green line).


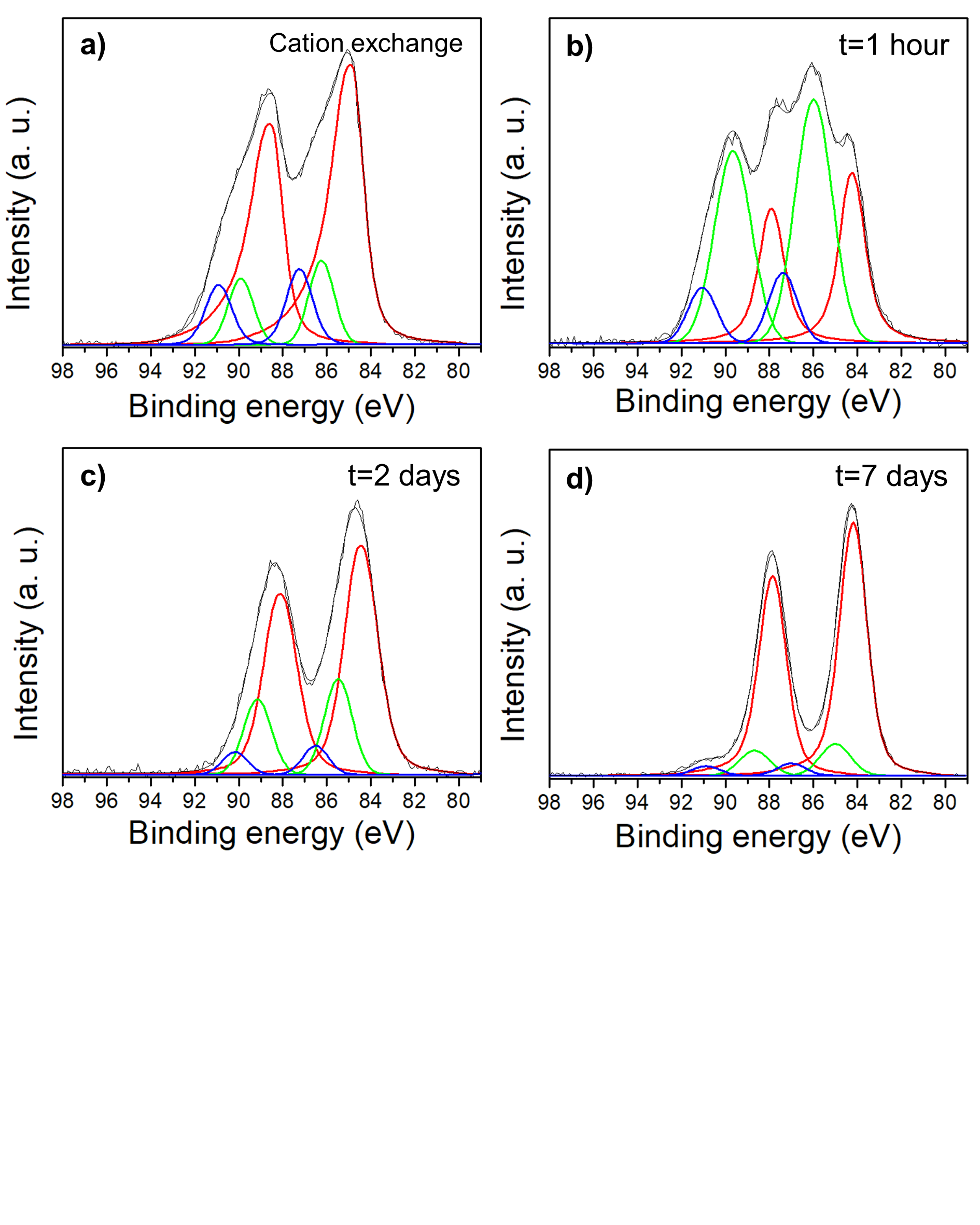


**Figure S3**. Au4f (Au^0^, red line; Au^+^, green line; Au^3+^, blue line) (a) XPS spectrum 1 h after cation exchange and centrifugation. Au4f XPS spectra of the synthesis of the UC_Tm_@AuNC at different reaction times: (b) 1 hour, (c) 2  and (d) 7 days after addition of GSH. XPS was performed in the solid pellet after centrifuging the dispersion at the indicated times after GSH addition.

**Table S1.** XPS ratio of Au^0^, Au^+^ y Au^3+^ at the indicated times after the GSH addition.

|  |  | **Ratio (%)** | | |
| --- | --- | --- | --- | --- |
| **Colloid** | **Synthesis step** | **Au^0^** | **Au^+^** | **Au^3+^** |
| UC_Tm_@AuNC | Cation exchange + 1 hour | 73.4 | 13.5 | 13.1 |
|  | GSH addition + 1 hour | 33.6 | 54.3 | 12.1 |
|  | GSH addition + 2 days | 71.7 | 22.0 | 6.3 |
|  | GSH addition + 7 days | 86.0 | 10.3 | 3.7 |
| AuNC | GSH addition + 7 days | 80.5 | 19.5 |  |


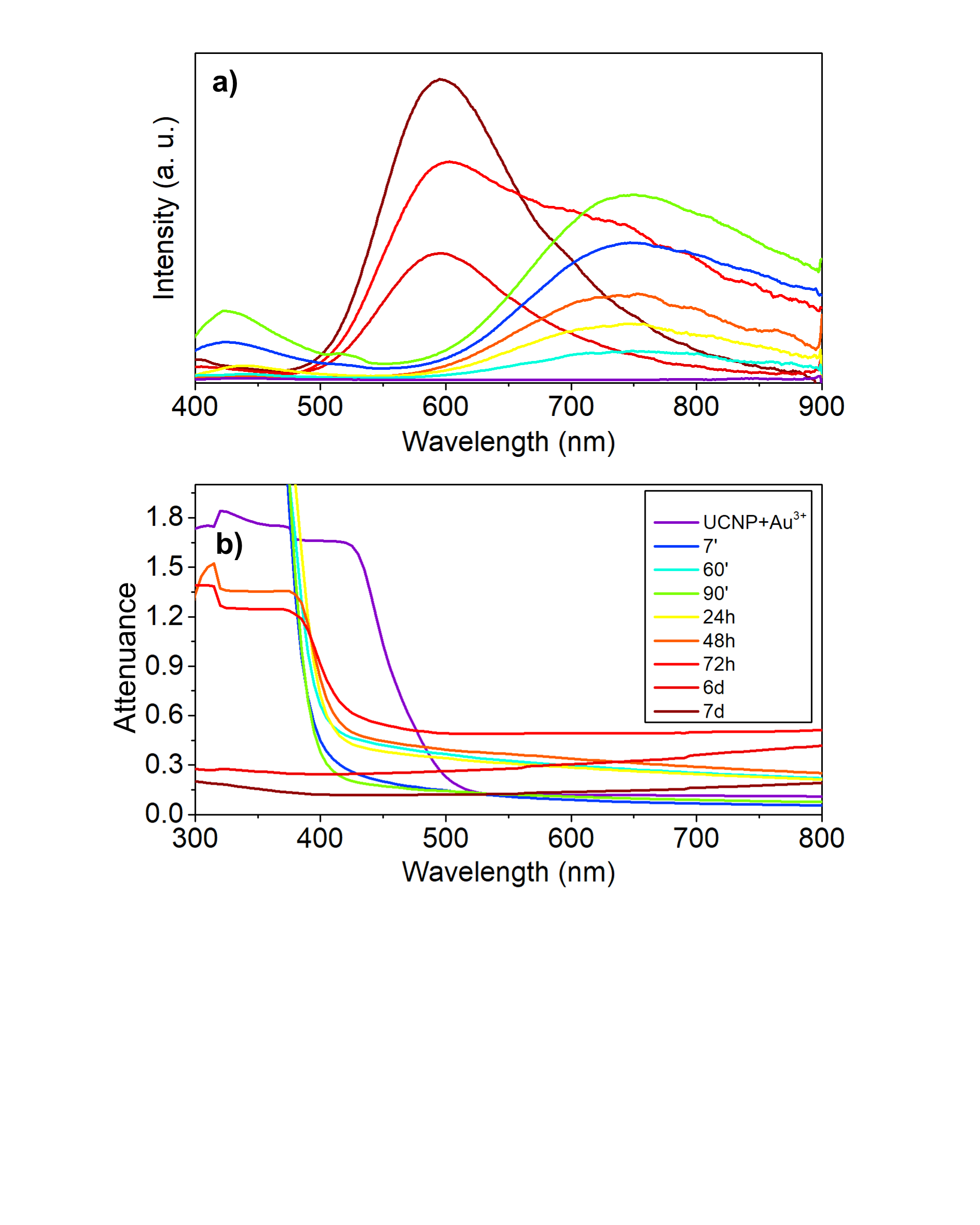


**Figure S4.** (a) Emission and (b) attenuance spectra of the evolution of the UC_Tm_@AuNC synthesis at the indicated times after GSH addition. The samples used to record the spectra were not subjected to purification steps.


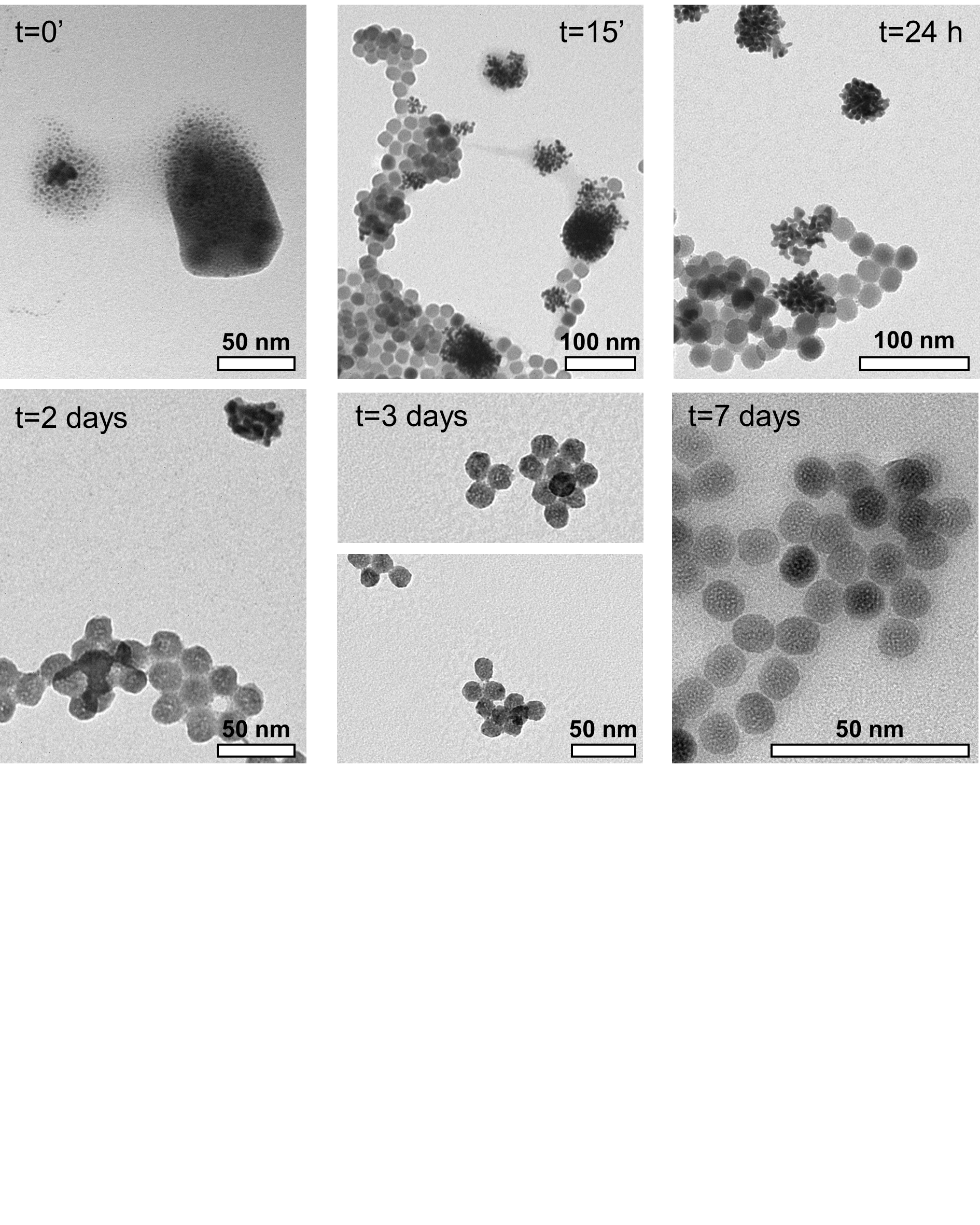


**Figure S5**. TEM images of the evolution of the synthesis of UC_Tm_@AuNC. The samples used to take the images were not subjected to purification steps, except for the 7^th^ day sample.


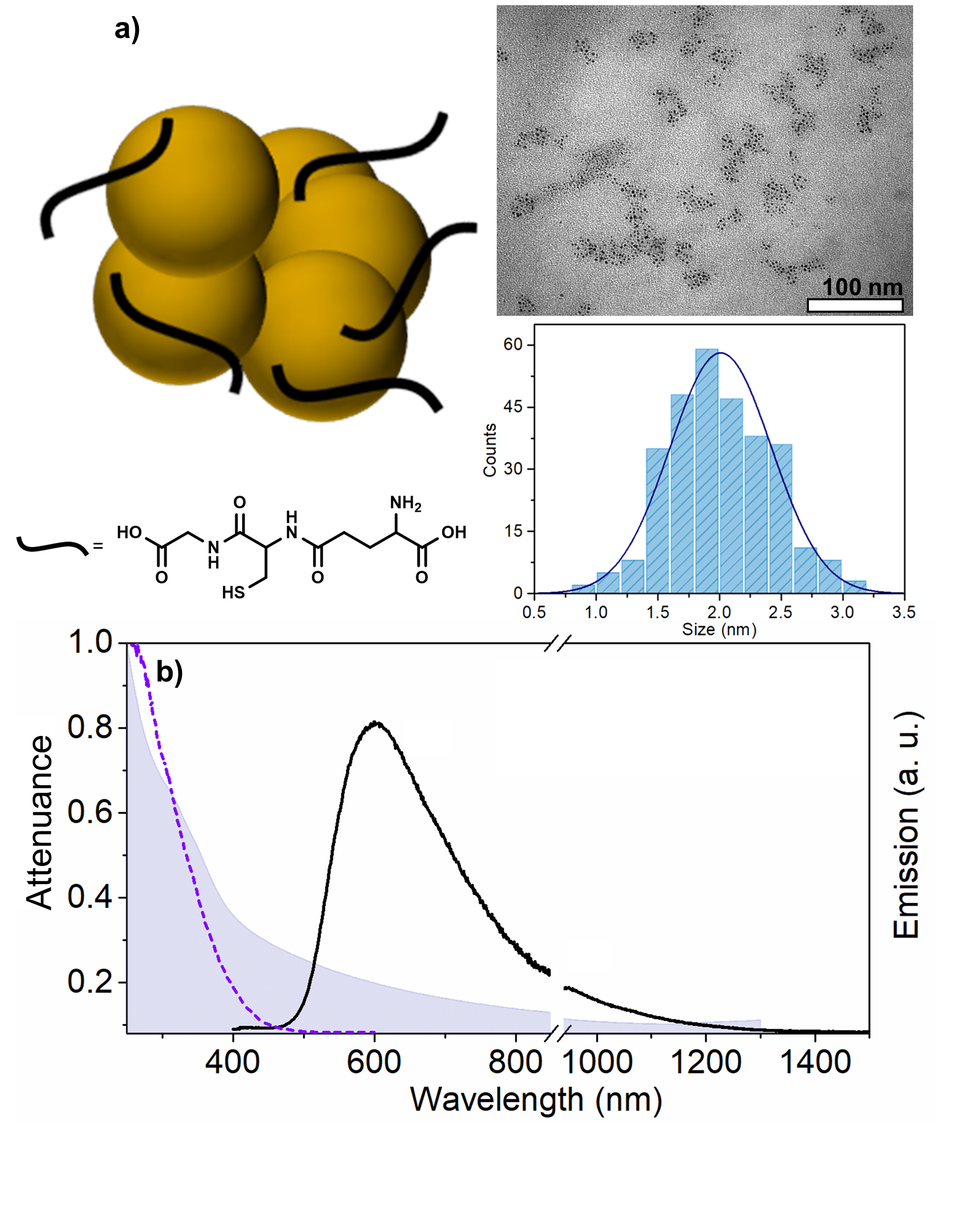


**Figure S6**. (a) Pictorial representation of AuNC. TEM image and size distribution of AuNC. (b) Attenuance spectrum (purple area), excitation spectrum (purple dashed line) and emission spectrum (black line) of AuNC (0.5 mg·mL^-1^) upon 350 nm excitation in D_2_O.


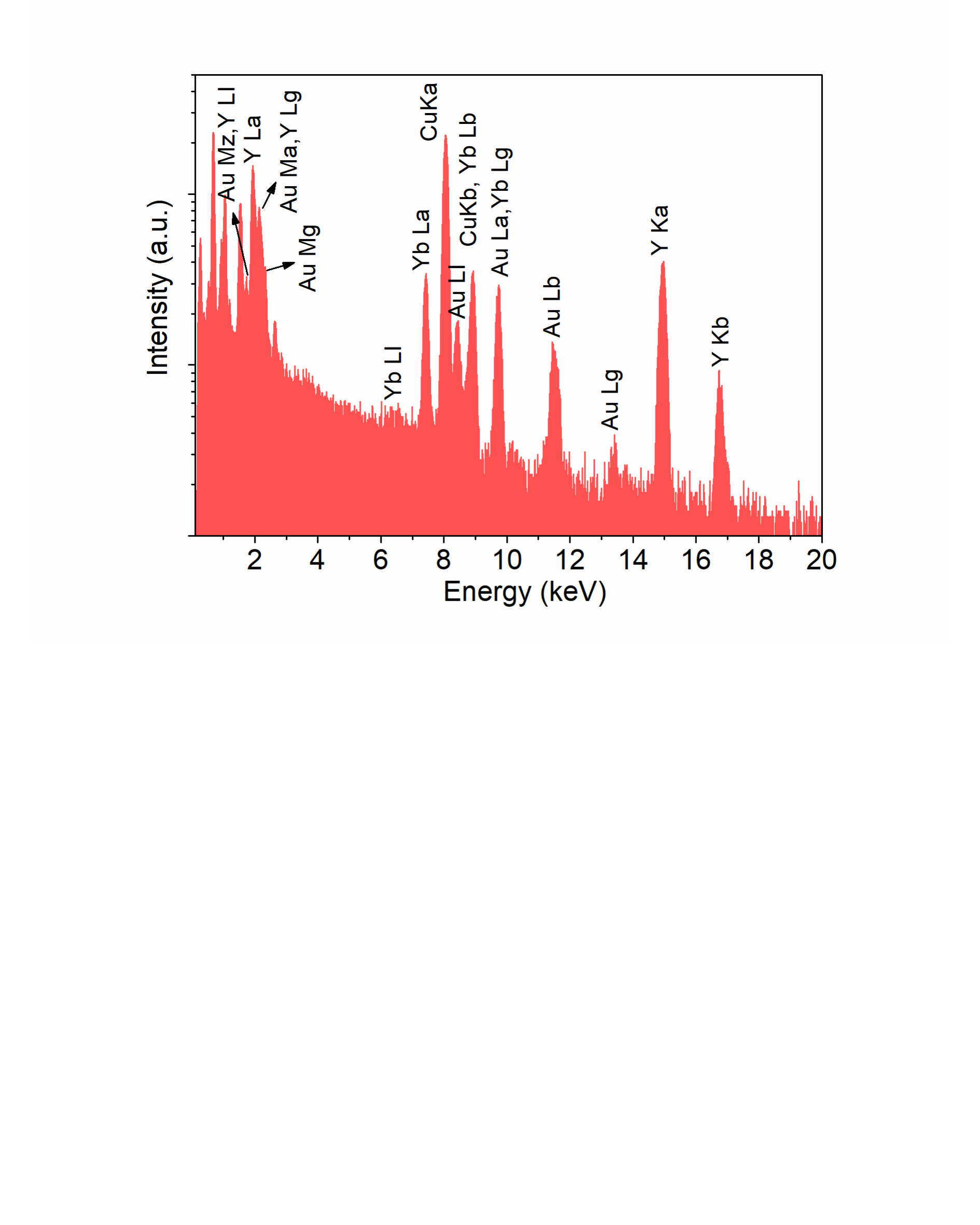


**Figure S7**. EDAX spectrum of UC_Tm_@AuNC.


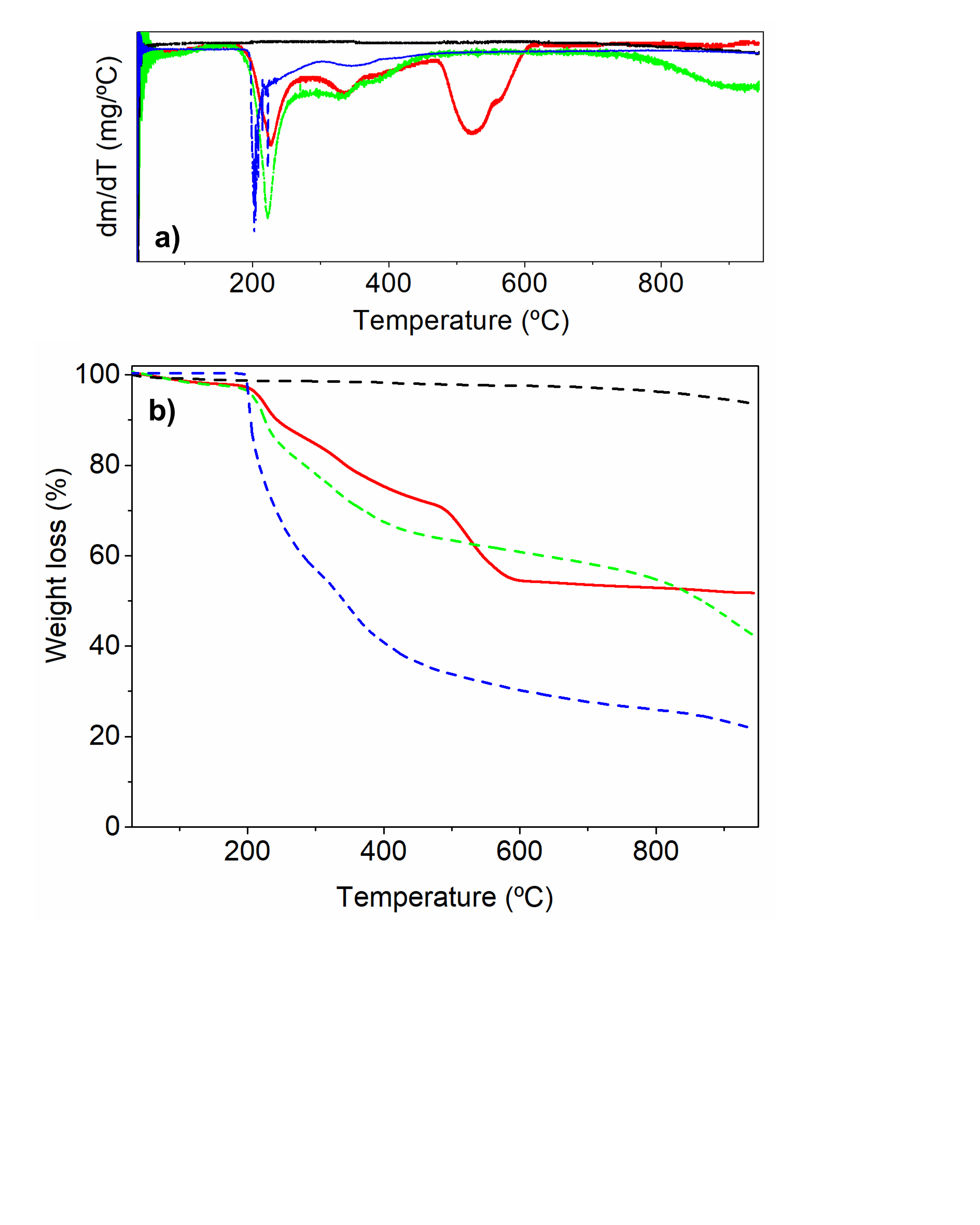


**Figure S8.** (a) First derivatives and (b) thermogravimetric analyses (TGA) of the UC_Tm_@AuNC NHS (red full line) and the references (dashed lines): UC_Tm,_ (black), GSH (blue) and AuNC (green) are included for comparison.





**Figure S9.** AuNC XPS spectra of Au4f (Au^0^, red line; Au^+^, green line).





**Figure S10**. UC_Tm_@AuNC XPS spectra of S2p (light blue line) and Y3d (orange line).


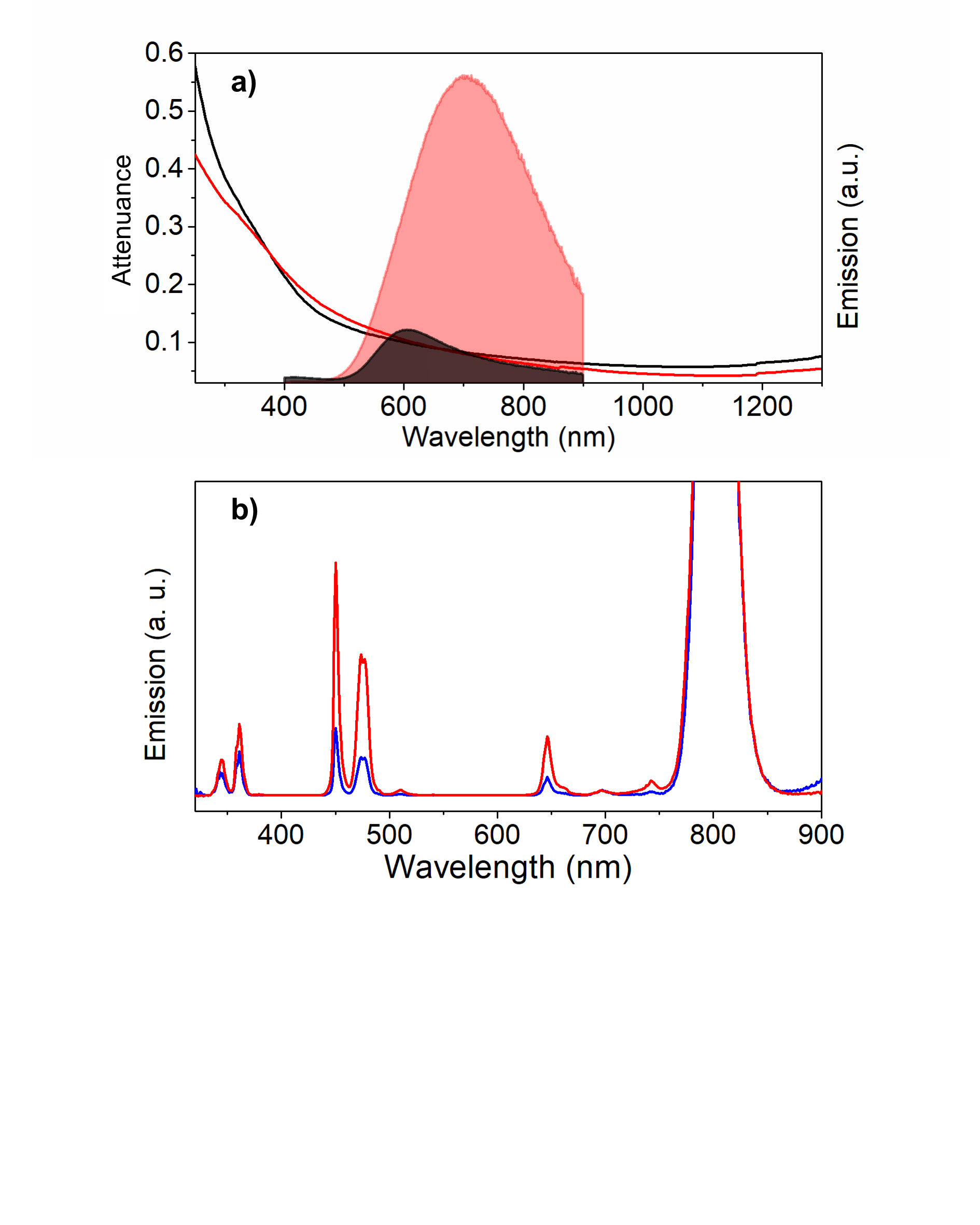


**Figure S11.** (a) Attenuance spectrum (line) and emission spectrum (colored area) of AuNC (black) and UC_Tm_@AuNC (red) upon 350 nm excitation in H_2_O (0.5 mg·mL^-1^). (b) Emission spectrum of UC_Tm_ (blue line) and UC_Tm_@AuNC (red line) upon 980 nm excitation in H_2_O (0.5 mg·mL^-1^).





**Figure S12.** Excitation spectra of UC_Tm_ (red line) and UC_Tm_@AuNC (black line) (λ_em_ 978 nm).


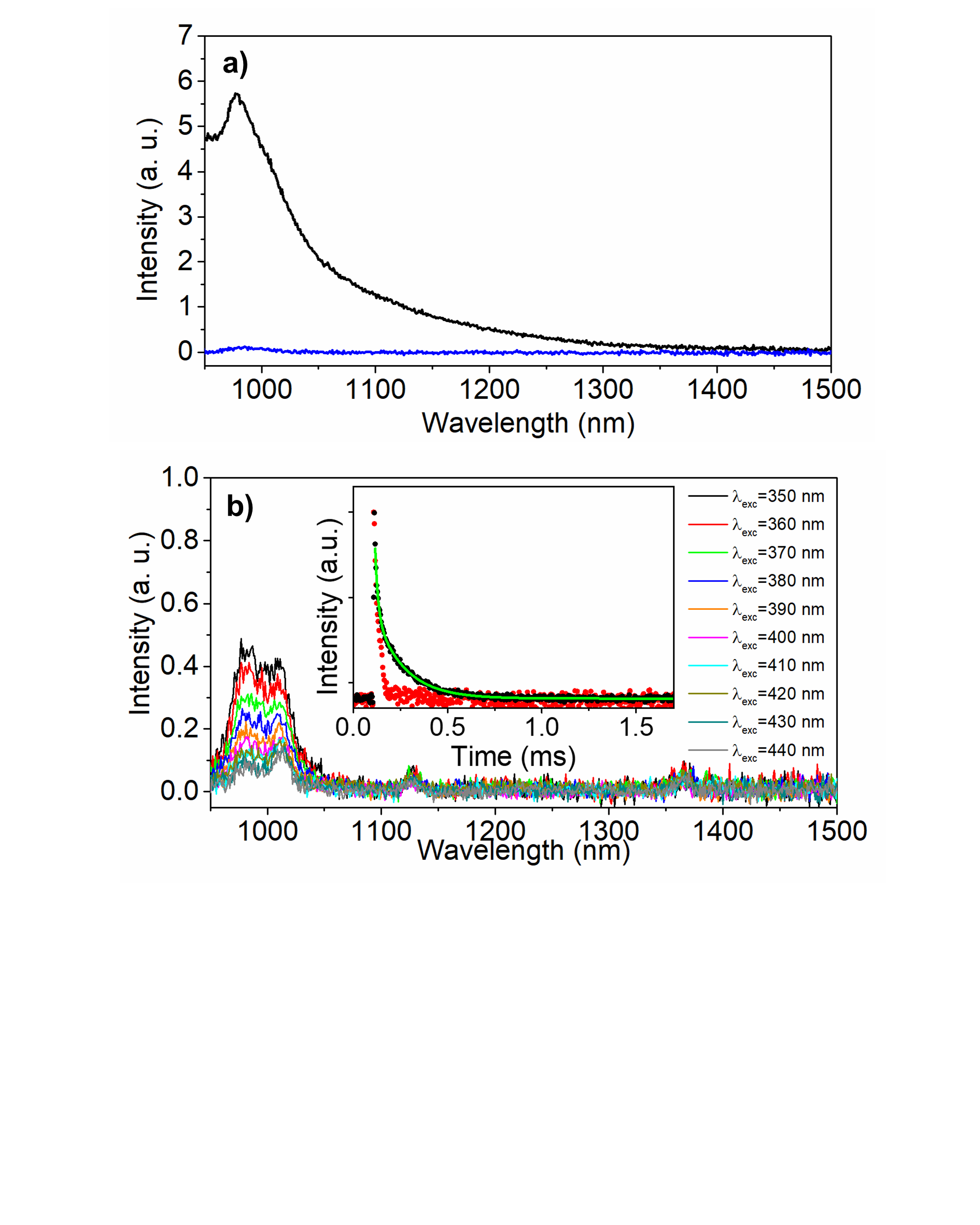


**Figure S13.** (a) Emission spectra of UC_Tm_ (blue line) and UC_Tm_@AuNC (black line) (λ_exc_ 350 nm). (b) Emission map (λ_exc_ 350-440 nm) of UC_Tm_. Inset: kinetic profile (black dots) and fitting (green line) of UC_Tm_ (λ_exc_ 350 nm; λ_em_ 1000 nm). The IRF was measured with the sample and is shown as red dots (λ_exc_ 350 nm; λ_em_ 950 nm).


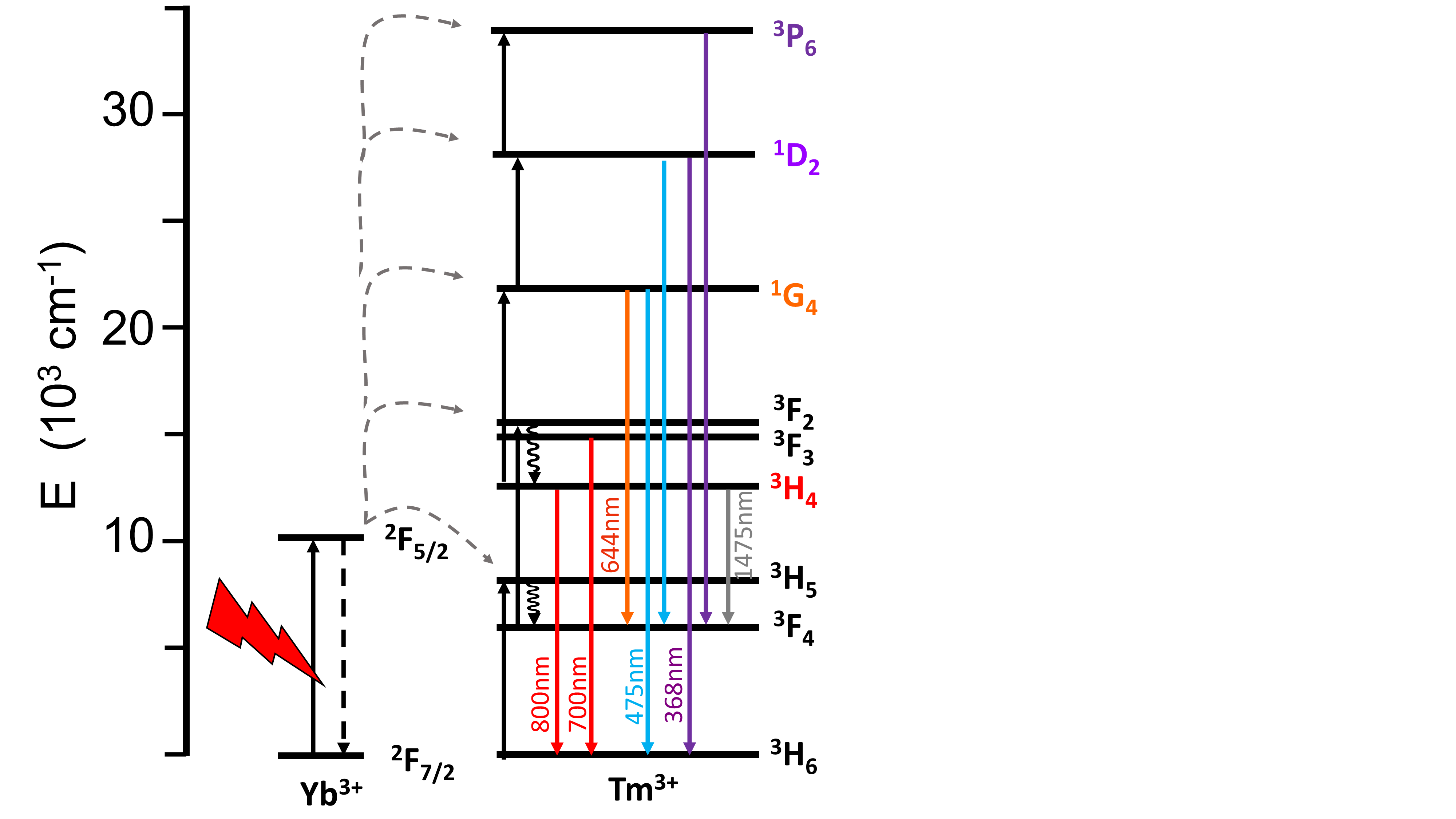


**Figure S14**. Energy level diagram and upconversion emission pathways (λ_exc_=980 nm) of NaYF_4_:Yb^3+^,Tm^3+^ LnNP.


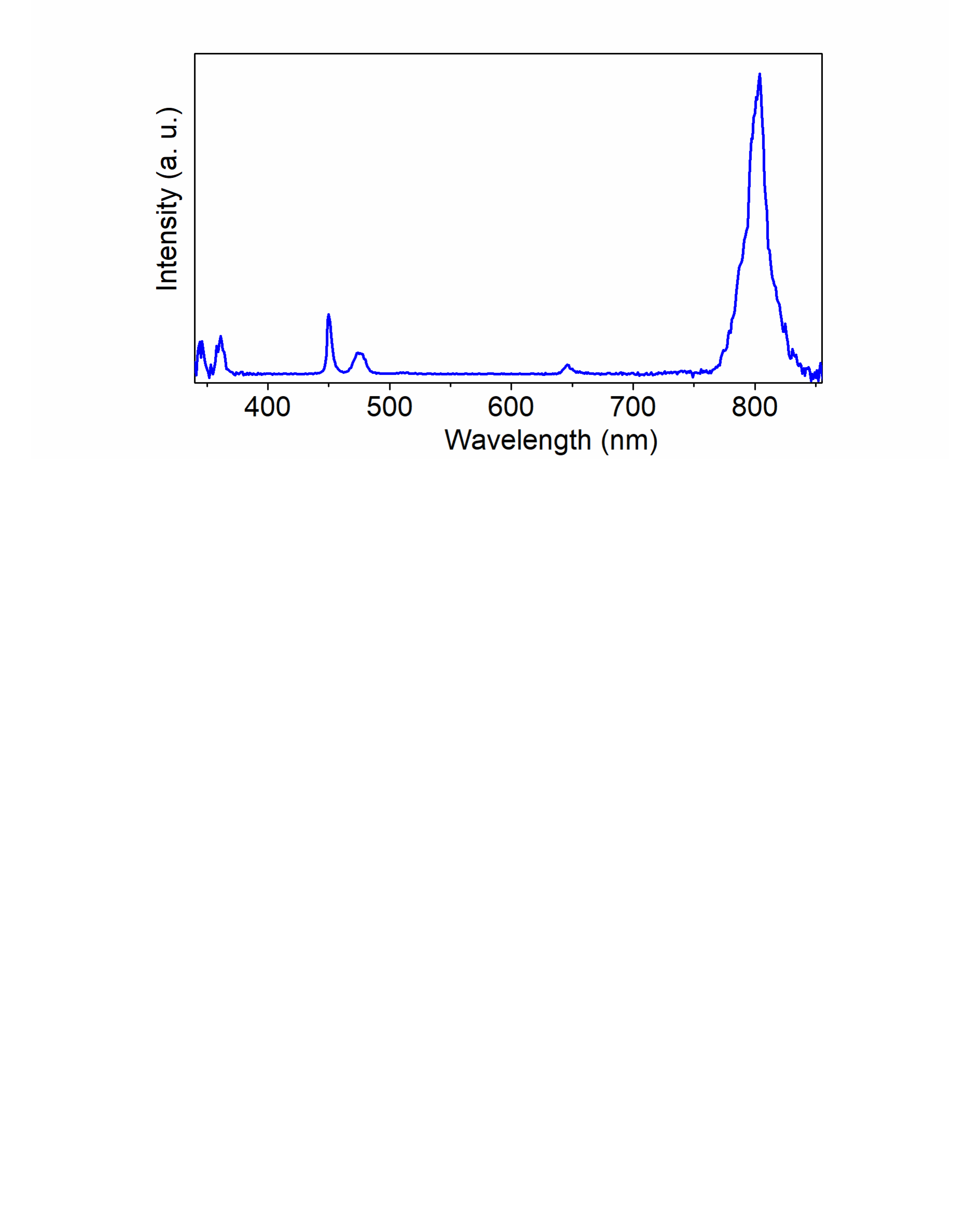


**Figure S15**. Emission spectra of UC_Tm_ (blue line) upon 980 nm excitation (PD=9 W·cm^-2^).





**Figure S16**. UCQY dependence with the excitation power density of UC_Tm_ (pink squares), UC_Tm_ after 1h of cation exchange with Au^3+^ (blue squares) and UC_Tm_@AuNC* (green squares).

* The absolute UCQY has been measured for solid UC_Tm_@AuNC. Emission of low-concentrated dispersions could not be detected, and concentrated off-white dispersions did not remain dispersed.





**Figure S17**. Kinetic profiles (dots) and fitting (line) at λ_exc_ 980 nm and λ_em_ 1000 nm of UC_Tm_ (red dots, black line), UC_Tm_ after 1h of cation exchange with Au^3+^ (green dots, blue line) and UC_Tm_@AuNC (black dots, red line).

**Table S2.** Yb^3+^ PL lifetimes of 0.5 mg/mL dispersions of UC_Tm_ and UC_Tm_@AuNC recorded upon 980 nm laser excitation.

| Sample | τ_PL,av_  (µs) |
| --- | --- |
| UC_Tm_ | 78.1±0.2 |
| UC_Tm_@AuNC | 198.5±1.3 |
| UC_Tm_ after 1h of cation exchange with Au^3+^ | 165.1±0.2 |


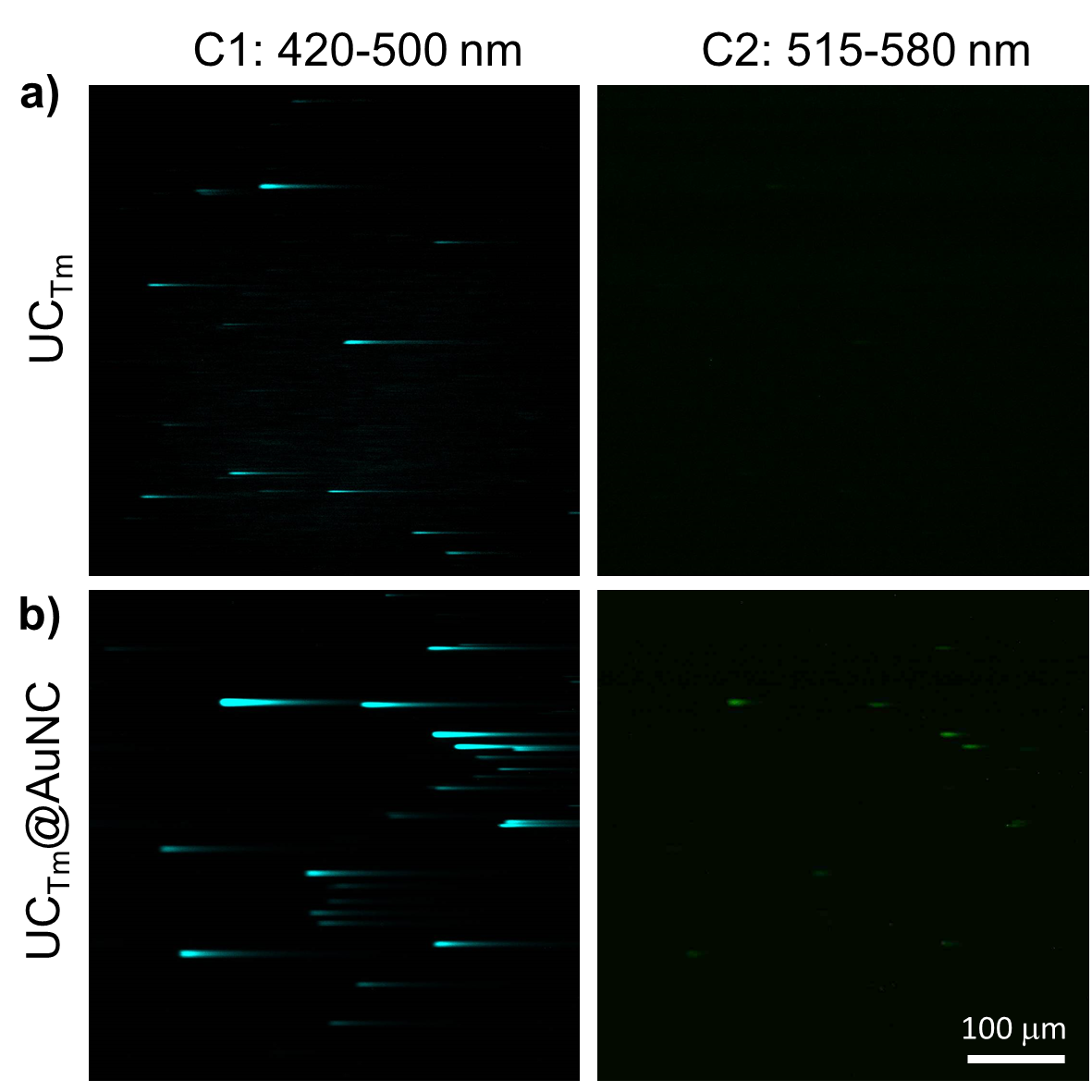


**Figure S18**. NIR-LSM images of (a) UC_Tm_ and (b) UC_Tm_@AuNC obtained in the (left) detection channel 1 and (right) detection channel 2 (λ_exc_ 975 nm, dwell time: 8 μs·pixel^-1^; F 59 J·cm^-2^). 100 μm scale bar applies for all the images.


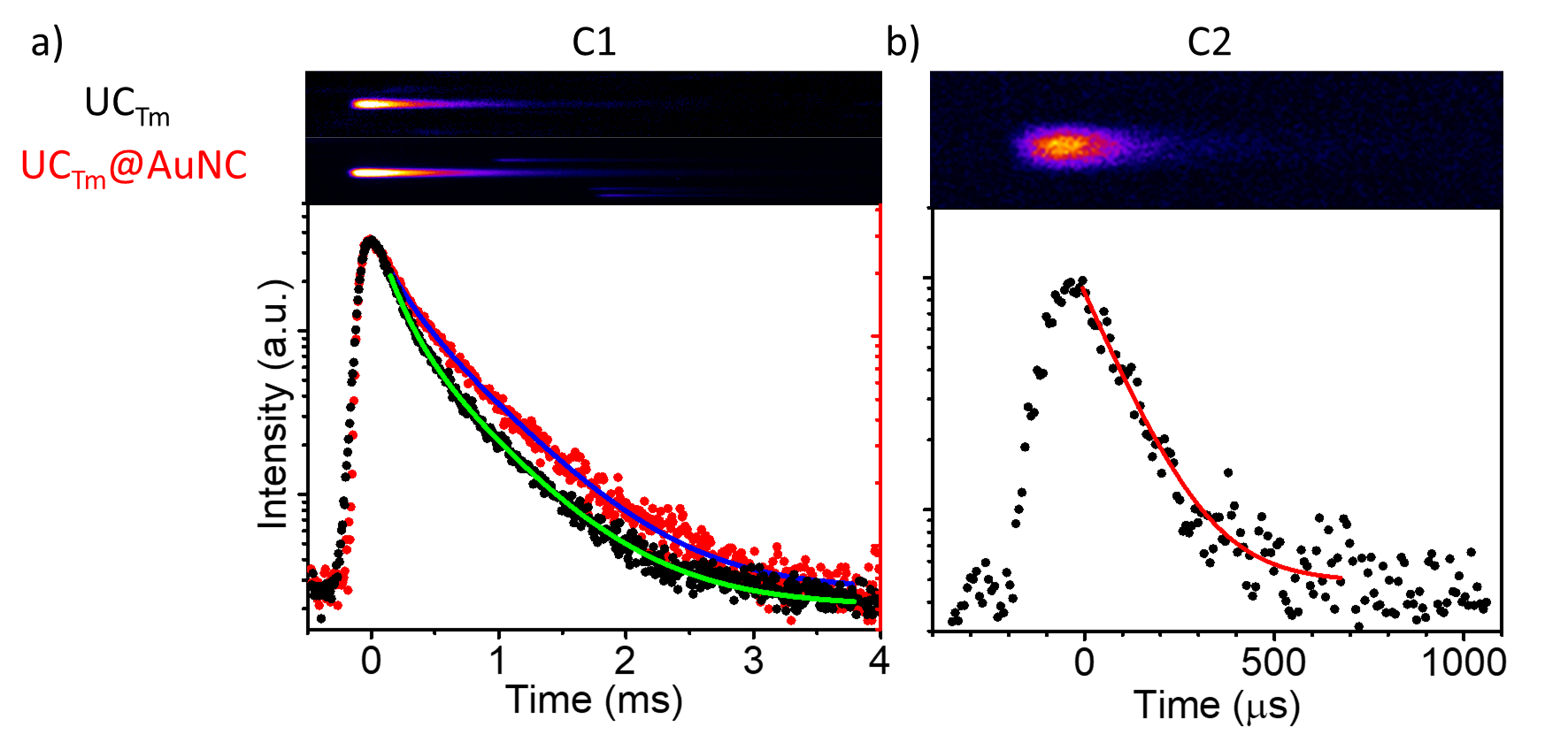


**Figure S19.** (a) Kinetic profiles and fitting of the Tm^3+^ upconversion emission (C1, λ_em_=420‑500 nm) of UC_Tm_ (black dots) and UC_Tm_@AuNC (red dots; λ_exc_ 975 nm; dwell time: 8 μs·pixel^-1^; F 59 J·cm^-2^). (b) Kinetic profile and fitting of the sensitized emission of the AuNC emission (C2, λ_em_=515-580 nm) of UC_Tm_@AuNC (λ_exc_ 975 nm; dwell time: 8 μs·pixel^-1^; F 59 J·cm^-2^).

**Table S3.** Fitting parameters of the kinetics obtained for several tails observed in the NIR-LSM images for the indicated samples.

| Detection channels | Colloid | *τ*_PL,av_(μs) | *τ*_PL,1_(μs) | A1(%) | *τ*_PL,2_ (μs) | A2(%) |
| --- | --- | --- | --- | --- | --- | --- |
| C1  (420-500 nm) | AuNC | 514 ± 20 | 165 ± 23 | 23 | 618 ± 25 | 77 |
|  | UC_Tm_@AuNC | 494 ± 19 | 195 ± 35 | 23 | 585 ± 34 | 77 |
| C2  (515-580 nm) | UC_Tm_@AuNC |  | 124 ± 14 |  |  |  |


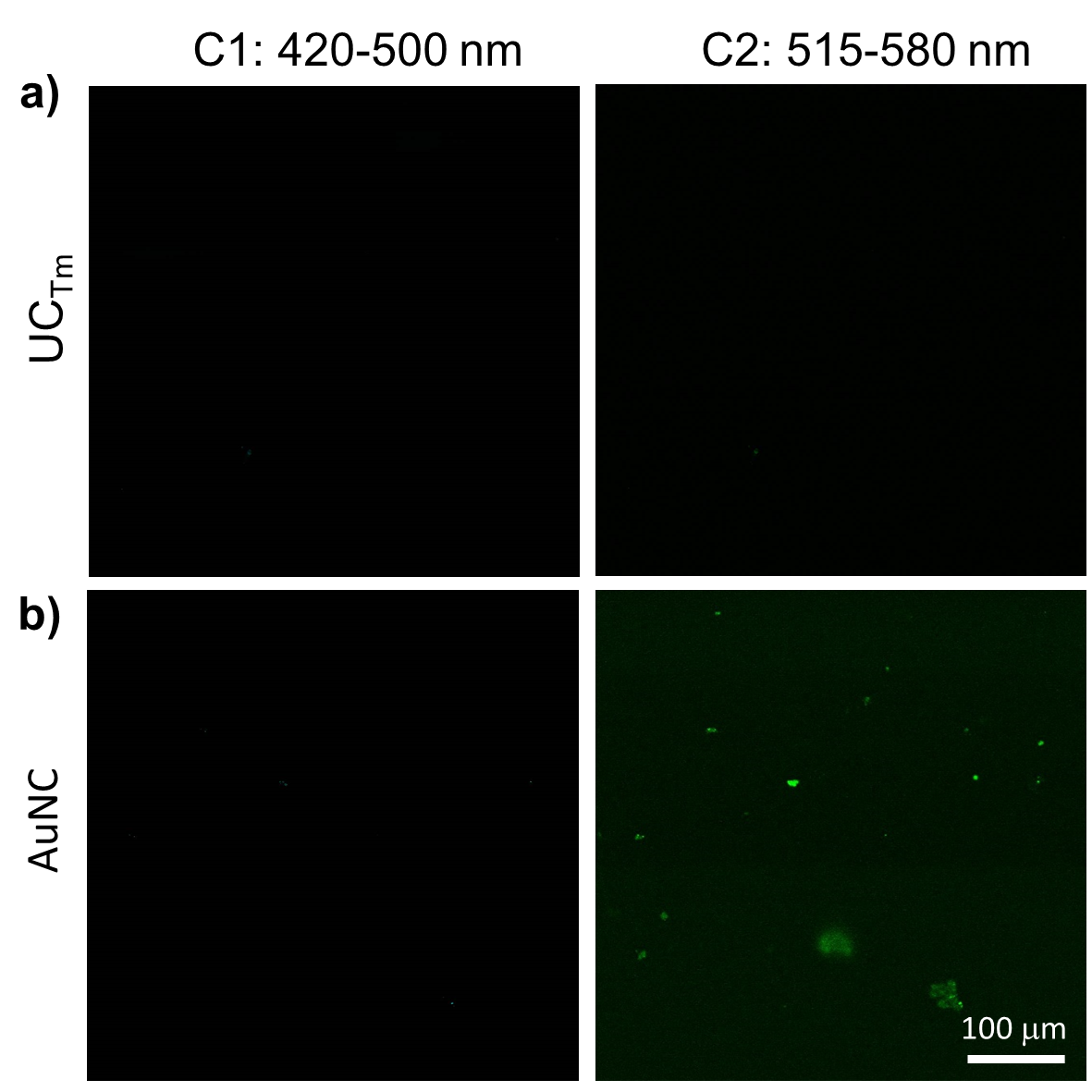


**Figure S20**. NIR-LSM images of (a) UC_Tm_ and (b) AuNC obtained in the (left) detection channel 1 and (right) detection channel 2 (λ_exc_ 800 nm, dwell time: 4 μs·pixel^-1^; F 27 J·cm^-2^). 100 μm scale bar applies for all the images.





**Figure S21**. Cell viability (%) of HeLa cells according to dehydrogenase activity after incubation with different concentrations of UC_Tm_@AuNC (red bar), UC_Tm_ (purple bar), and AuNC (green bar). Cells without exposure to nanoparticles represent 100 %. Negative control (NC, blue bar). Positive control (PC, grey bar). Values are shown as mean ± standard deviation.

REFERENCES

(1) Li, Z.; Zhang, Y. An Efficient and User-Friendly Method for the Synthesis of Hexagonal-Phase NaYF_4_:Yb, Er/Tm Nanocrystals with Controllable Shape and Upconversion Fluorescence. *Nanotechnology* **2008**, *19* (34), 15–20. https://doi.org/10.1088/0957-4484/19/34/345606.

(2) Estebanez, N.; González-Béjar, M.; Pérez-Prieto, J. Polysulfonate Cappings on Upconversion Nanoparticles Prevent Their Disintegration in Water and Provide Superior Stability in a Highly Acidic Medium. *ACS Omega* **2019**, *4* (2), 3012–3019. https://doi.org/10.1021/acsomega.8b03015.

(3) Francolon, N.; Boyer, D.; Leccia, F.; Jouberton, E.; Walter, A.; Bordeianu, C.; Parat, A.; Felder-Flesch, D.; Begin-Colin, S.; Miot-Noirault, E.; Chezal, J. M.; Mahiou, R. Preparation of Core/Shell NaYF_4_:Yb,Tm@dendrons Nanoparticles with Enhanced Upconversion Luminescence for in Vivo Imaging. *Nanomedicine Nanotechnology, Biol. Med.* **2016**, *12* (7), 2107–2113. https://doi.org/10.1016/j.nano.2016.05.020.

(4) Tang, J.; Lei, L.; Feng, H.; Zhang, H.; Han, Y. Preparation of K+-Doped Core-Shell NaYF_4_:Yb, Er Upconversion Nanoparticles and Its Application for Fluorescence Immunochromatographic Assay of Human Procalcitonin. *J. Fluoresc.* **2016**, *26* (6), 2237–2246. https://doi.org/10.1007/s10895-016-1919-8.

(5) Chen, C.; Kang, N.; Xu, T.; Wang, D.; Ren, L.; Guo, X. Core-Shell Hybrid Upconversion Nanoparticles Carrying Stable Nitroxide Radicals as Potential Multifunctional Nanoprobes for Upconversion Luminescence and Magnetic Resonance Dual-Modality Imaging. *Nanoscale* **2015**, *7* (12), 5249–5261. https://doi.org/10.1039/c4nr07591a.

(6) Wilhelm, S.; Kaiser, M.; Würth, C.; Heiland, J.; Carrillo-Carrion, C.; Muhr, V.; Wolfbeis, O. S.; Parak, W. J.; Resch-Genger, U.; Hirsch, T. Water Dispersible Upconverting Nanoparticles: Effects of Surface Modification on Their Luminescence and Colloidal Stability. *Nanoscale* **2015**, *7* (4), 1403–1410. https://doi.org/10.1039/c4nr05954a.

(7) Xiao, Q.; Ji, Y.; Xiao, Z.; Zhang, Y.; Lin, H.; Wang, Q. Novel Multifunctional NaYF_4_:Er^3+^,Yb^3+^/PEGDA Hybrid Microspheres: NIR-Light-Activated Photopolymerization and Drug Delivery. *Chem. Commun.* **2013**, *49* (15), 1527–1529. https://doi.org/10.1039/c2cc37620b.

(8) Dong, A.; Ye, X.; Chen, J.; Kang, Y.; Gordon, T.; Kikkawa, J. M.; Murray, C. B. A Generalized Ligand-Exchange Strategy Enabling Sequential Surface Functionalization of Colloidal Nanocrystals. *J. Am. Chem. Soc.* **2011**, *133* (4), 998–1006. https://doi.org/10.1021/jal08948z.

(9) Muhr, V.; Würth, C.; Kraft, M.; Buchner, M.; Baeumner, A. J.; Resch-Genger, U.; Hirsch, T. Particle-Size-Dependent Förster Resonance Energy Transfer from Upconversion Nanoparticles to Organic Dyes. *Anal. Chem.* **2017**, *89* (9), 4868–4874. https://doi.org/10.1021/acs.analchem.6b04662.

(10) Jones, C. M. S.; Gakamsky, A.; Marques-Hueso, J. The Upconversion Quantum Yield (UCQY): A Review to Standardize the Measurement Methodology, Improve Comparability, and Define Efficiency Standards. *Sci. Technol. Adv. Mater.* **2021**, *22* (1), 810–848. https://doi.org/10.1080/14686996.2021.1967698.
